# A point mutation in the photosystem II protein PsbW disrupts thylakoid organization and alters starch granule formation

**DOI:** 10.1093/plphys/kiaf206

**Published:** 2025-05-29

**Authors:** Theresa E Ilse, Hongyuan Zhang, Arvid Heutinck, Chun Liu, Simona Eicke, Mayank Sharma, Barbara Pfister, Diana Santelia, Samuel C Zeeman

**Affiliations:** Institute of Molecular Plant Biology, ETH Zurich, Zurich 8092, Switzerland; Institute of Integrative Biology, ETH Zurich, Zurich 8092, Switzerland; Institute of Molecular Plant Biology, ETH Zurich, Zurich 8092, Switzerland; Institute of Molecular Plant Biology, ETH Zurich, Zurich 8092, Switzerland; Institute of Molecular Plant Biology, ETH Zurich, Zurich 8092, Switzerland; Institute of Molecular Plant Biology, ETH Zurich, Zurich 8092, Switzerland; Institute of Molecular Plant Biology, ETH Zurich, Zurich 8092, Switzerland; Institute of Integrative Biology, ETH Zurich, Zurich 8092, Switzerland; Institute of Molecular Plant Biology, ETH Zurich, Zurich 8092, Switzerland

## Abstract

Chloroplast thylakoid membranes are the sites of the light reactions of photosynthesis. They are also thought to influence starch granule biogenesis via the thylakoid-anchored protein MAR-BINDING FILAMENT-LIKE PROTEIN 1 (MFP1), but mechanistic understanding is scarce. Here, we report an Arabidopsis (*Arabidopsis thaliana*) mutant affected in PHOTOSYSTEM II REACTION CENTER W (PsbW), an integral thylakoid membrane protein associated with photosynthetic complexes of PSII. This mutant (*psbw*-2) was identified in a large-scale mutant screen designed to find proteins that regulate starch granule shape and size because it produces an excessive number of small, irregularly-shaped starch granules. The mutation in *psbw-2* causes a glycine-to-arginine substitution in PsbW's transmembrane helix. The resulting PsbW^G107R^ protein remains membrane-associated but has lost its ability to stabilize PSII supercomplexes. In addition, the transgenic expression of this mutated version results in abnormal thylakoid membranes that have drastically enlarged luminal spaces and no longer-form distinct grana stacks, leading to reduced plant growth and impaired photosynthesis. These effects increase with PsbW^G107R^ expression levels but are not observed in the *psbw* knockout mutant, suggesting that PsbW^G107R^ has acquired an aberrant function. We analyzed *psbw*-2 mutants also lacking either MFP1 or STARCH SYNTHASE 4 (SS4), a key factor involved in granule initiation and growth. These data suggest that thylakoid distortion is caused by the membrane insertion of PsbW^G107R^, which in turn affects the initiation and growth of starch granules. Our results reaffirm the link between the thylakoid membrane system and starch formation and highlight the importance of proper thylakoid architecture for plant fitness.

## Teaser

A mutation in a photosynthesis-related membrane protein disrupts chloroplast structure, revealing a link between chloroplast internal membrane architecture and the formation of starch granules.

## Introduction

Chloroplasts, the plant cell organelles originating from a cyanobacterial endosymbiont, are responsible for photosynthesis and the synthesis of many key metabolites, including the storage carbohydrate starch. The chloroplast double envelope surrounds an aqueous stroma and a network of glycolipid-rich thylakoid membranes containing proteins that carry out the light reactions of photosynthesis. The thylakoids enclose a single, interconnected luminal space and are organized into cylindrical stacks of 5 to 20 appressed thylakoids (grana) connected by stromal lamellae (Mustardy 2003; Shimoni et al. 2005; Pribil et al. 2014).

Recently, the early stages of chloroplast development in Arabidopsis (*Arabidopsis thaliana*) were described in cotyledons using 3D electron tomography (Liang et al. 2018). Upon illumination, tubule vesicular pro-thylakoids form from the proplastid inner envelope membrane, then mature into sheet-like pre-granal thylakoids, as photosystem II (PSII) complexes start to assemble. Subsequently, pro-grana stacks form and light-harvesting complex II (LHCII), ATP synthase, and the cytochrome b_6_f complex (Cyt b_6_f) accumulate. Photosystem I (PSI) complexes assemble last and the thylakoid network interconnects via lateral fusion of the grana stacks. Relatively little is known about the mechanisms that control this intricate thylakoid development.

The stroma-exposed thylakoid membranes differ from the appressed membranes in terms of their protein composition, particularly in the distribution of the photosynthetic complexes. For example, PSII and LHCII are found in the appressed grana stacks, while PSI with its light-harvesting complex (LHCI) and the ATP synthase are mainly present in the stromal lamellae (Pribil et al. 2014). The non-uniform distribution of these complexes is suggested to enhance light harvesting and optimize the light reactions of photosynthesis by regulating the cyclic and linear electron flow (Andersson and Anderson 1980; Albertsson 2001; Johnson et al. 2014; Pribil et al. 2014; Wietrzynski et al. 2020). Other proteins are also non-uniformly distributed, such as MAR-BINDING FILAMENT-LIKE PROTEIN 1 (MFP1) (Jeong et al. 2003). This protein contains a transmembrane thylakoid anchor and localizes in distinct puncta that direct the initiation of starch granule biogenesis in chloroplasts (Sharma et al. 2024).

Starch comprises a mixture of 2 glucose polymers, amylopectin and amylose and is deposited as insoluble, semi-crystalline granules. In leaf chloroplasts, so-called transitory starch is a major product of daytime photosynthesis serving as a short-term carbohydrate storage, which fuels metabolic processes at night when photosynthesis is not possible (reviewed e.g. in Zeeman et al. 2010; Goren et al. 2018; Smith and Zeeman 2020). In contrast, storage starch synthesized in amyloplasts of heterotrophic tissues (e.g. roots, tubers, seeds) serves as a long-term carbohydrate storage. Storage starch has obvious nutritive importance but also has interesting physico-chemical properties, rendering it a valuable renewable raw material for industry (Röper 2002; Jobling 2004; Chen et al. 2021).

The size and morphology of granules influence starch properties, and there is remarkable variation in these traits between species but also between tissues of the same species (Chen et al. 2021). While storage starch granules can reach up to 50 *µ*m in diameter or more (Tester et al. 2004; Chen et al. 2021), transitory starch granules are more similar between species and smaller (<2 *µ*m) (Grange et al. 1989; Cabálková et al. 2008). Sections of Arabidopsis mesophyll cell chloroplasts typically reveal 5 to 7 lenticular starch granules embedded between thylakoid membranes in so-called stromal pockets (Crumpton-Taylor et al. 2012; Bürgy et al. 2021). Isotope labelling revealed that these granules grow anisotropically (Bürgy et al. 2021), explaining their characteristic flattened spheroid morphology. Although the enzymes involved in starch biosynthesis and degradation have been studied extensively (see Pfister and Zeeman [2016] and Smith et al. [2005]), less is known about the factors controlling granule number, size, and their growth patterns.

Recently, several Arabidopsis mutants were identified with alterations in the size and number of starch granules. While their overall starch contents are often similar to the wild type, they synthesize fewer, larger granules per chloroplast, likely because they lack proteins needed for normal granule initiation (Seung et al. 2018; Seung and Smith 2019; Abt et al. 2020). The emerging picture for transitory starch granule initiation points to a pathway centered around the glycosyltransferase STARCH SYNTHASE 4 (SS4), which is proposed to elaborate soluble glucan primers into substrates suitable for the rest of the starch biosynthetic machinery, thereby priming insoluble granule formation (Roldán et al. 2007; Lu et al. 2018). Suitable malto-oligosaccharide (MOS) primers are probably delivered to SS4 via PROTEIN TARGETING TO STARCH proteins (PTST2 and PTST3), which possess carbohydrate-binding modules (Seung et al. 2017). PTST2 physically interacts with both SS4 and its own homolog PTST3 and has been suggested to bind long MOS adopting a helical conformation. PTST2 also physically interacts with MFP1, which thereby directs the initiation process to distinct stromal sites adjacent to the thylakoid membranes (Seung et al. 2018; Sharma et al. 2024).

A few mutants are known that also exhibit different granule shapes, the most prominent example being *ss4* itself; in addition to producing fewer and larger granules, its granules are near spherical instead of lenticular due to the loss of the anisotropic growth pattern (Roldán et al. 2007; Lu et al. 2018; Bürgy et al. 2021). Mutants lacking STARCH SYNTHASE 2 (SS2), a starch synthase involved in amylopectin chain elongation, also have irregularly shaped granules, probably due to the change in amylopectin structure (Zhang et al. 2008; Szydlowski et al. 2011; Pfister et al. 2014). Interestingly, the Arabidopsis *fzo-like* (*fzl*) mutant, which is deficient in a protein involved in thylakoid organization, also has an abnormal starch granule shape. This mutant produces fewer grana and its stromal lamellae are disorganized, especially around the starch granules, which consequently develop uneven surfaces, suggesting that the thylakoids also influence starch granule size and shape (Esch et al. 2022).

Here, we report a mutant identified in a genetic screen designed to discover additional factors influencing starch granule morphology. This mutant (*psbw*-2) shows increased numbers of granules per chloroplast with aberrant morphology. Surprisingly, this phenotype is caused by a mis-sense mutation in the *PHOTOSYSTEM II REACTION CENTER W* (*PsbW*) gene, encoding an integral membrane protein that is part of the photosystem II supercomplexes (Shi et al. 2000; Thidholm et al. 2002). The single amino acid change in PsbW's single transmembrane helix causes severe disorganization of the thylakoid membranes, with defects in photosynthesis, swelling of the lumen and abnormal stromal pocket shape—phenotypes not seen when the PsbW protein is missing altogether. This supports the idea that the architecture of the thylakoid membrane system influences starch granule formation.

## Results

### Identification of a *PsbW* mutant with altered granule morphology

**Figure 1.**
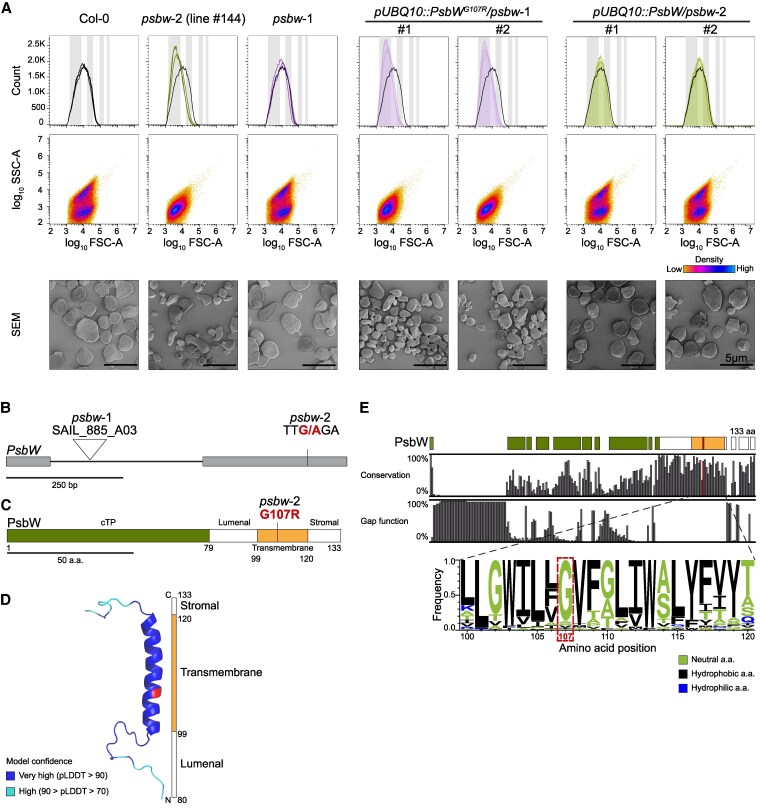
Starch granule phenotype of Arabidopsis *psbw* mutants and wild type (Col-0) and structure and conservation of PsbW. **A)** Analysis of starch granule morphology of starch from leaves harvested at the end of day. The *psbw*-2 (line #144) plants were analyzed in the M_4_ generation. The #1 and #2 lines of the complementation lines indicate two independent T_3_ generation lines. Top panels: Histograms of granule size distribution showing the forward scatter (FSC-A; indicating granule size) and granule count (*n* = 3 plants; 100,000 particles measured per replicate). Grey bars indicate the size ranges 0.1–0.5 *μ*m, 1–2 *μ*m, 4–6 *μ*m, and 8–10 *μ*m (left to right). The black lines show the representative wild-type sample for comparison. Middle panels: Scatter plot of sideward scatter (SSC-A) against forward scatter (FSC-A) indicating the granule shape. Results were consistent across 3 biological replicates; representative plots are shown. Lower panels: Scanning electron microscopy (SEM) of purified starch granules. Scale bar unit is shown in the last image of the row and applies to all images in that row. **B)** The Arabidopsis *PsbW* gene model (AT2G30570.1). Exons are represented as boxes and the intron as line. The triangle shows the site of T-DNA insertion of the *psbw*-1 knockout allele. The G to A substitution in the *psbw*-2 mutant is highlighted. bp, base pair. **C)** Protein domain organization of Arabidopsis PsbW. The transit peptide (cTP) is represented in green, luminal and stromal domains in white and the transmembrane domain in orange. The location of the amino acid exchange in *psbw*-2 is highlighted. a.a., amino acids. **D)** Alpha-Fold2 prediction of the PsbW structure. Cartoon representation of the folded protein without transit peptide (cTP); the mutated amino acid of *psbw*-2 is highlighted. Protein color shows the model confidence (pLDDT value). **E)** Conservation of the PsbW protein based on the alignment of >60 orthologous sequences (see phylogenetic tree in Supplementary Fig. S1). Colored boxes indicate the Arabidopsis PsbW domains (as in **C)**. The gap function plot shows that most of the lowly conserved regions occur only in a few putative orthologs. The Weblogo visualizes the conservation of PsbW's transmembrane domain (amino acid positions are based on Arabidopsis PsbW). a.a., amino acids.

We performed a mutagenesis screen in Arabidopsis to identify genes controlling starch granule morphology in chloroplasts. We treated wild-type (Col-0) Arabidopsis seeds with ethyl methanesulfonate (EMS) and screened the M_2_ generation for alterations in granule morphology using a high-throughput starch extraction protocol and flow cytometry (FC) analysis of starch granule size and shape (Thieme et al. 2023). In FC, the forward scatter (FSC-A) is proportional to the granule size, while the sideward scatter (SSC-A) provides information on particle granularity and can be used to infer granule shape. In a 2-dimensional scatter plot (FSC-A vs. SSC-A), a population of flat, discoid starch granules, such as from wild-type leaves, displays 2 density centers (Fig. 1A). In contrast, round granules (e.g. from *ss4* mutants) exhibit only one, compact density center (Thieme et al. 2023). Mutants with irregularly shaped granules generally have one diffuse density center (e.g. from *ss2* mutants).

We identified numerous mutants with altered starch morphology. In line #144, FC analysis indicated that the granules were smaller than wild-type granules and, based on the single diffuse density center in the scatter plot, abnormally shaped. Scanning electron microscopy (SEM) of extracted granules confirmed this phenotype (Fig. 1A). To identify the causative mutation, we crossed line #144 with the wild type and re-analyzed the F_2_ progeny for granule phenotypes by FC. We observed a ratio of wild-type to mutant phenotypes of 3.1:1 (in the 172 analyzed F_2_ plants), close to the 3:1 ratio expected for a monogenic recessive mutation. Pooled genomic DNA from all F_2_ plants showing the mutant phenotype was subjected to next-generation sequencing. Single nucleotide polymorphism (SNP) analysis suggested that the causative mutation was a missense mutation in the second exon of the *PsbW* gene (*At2g30570*; Fig. 1B), as this was the only SNP variant showing an allele frequency of 100% in the pooled mutant DNA (see Supplementary Table S1 for details). This mutation resulted in an amino acid change from glycine 107 to arginine (G107R) in the PsbW protein (Fig. 1C). We therefore renamed line #144 as *psbw*-2.

The mature PsbW is a small protein of only 54 amino acids (after cleavage of its 79 amino-acid chloroplast transit peptide; Fig. 1C) and is highly conserved. We found at least one putative PsbW ortholog in each of the 54 assessed species of land plants and green algae, but no orthologs in red algae, glaucophytes or cyanobacteria, consistent with previous reports (Shi and Schröder 2004; Grossman et al. 2019;  Supplementary Fig. S1). The only secondary structure predicted by Alphafold2 is the proteins’ hydrophobic α-helix, which integrates PsbW into the thylakoids, flanked by short luminal and stromal extensions without predicted secondary structure (Fig. 1D). The G107R substitution is within the transmembrane helix. Potential structural changes caused by single amino acid change are not reliably predicted by Alphafold2 (Pak et al. 2023), but it is likely that a non-conservative substitution would impact on the proteins behavior in the thylakoids. Notably, G^107^ is very well conserved and, in the few orthologs where it is substituted, it is with another neutral or hydrophobic amino acid. (Figure 1E).

**Figure 2.**
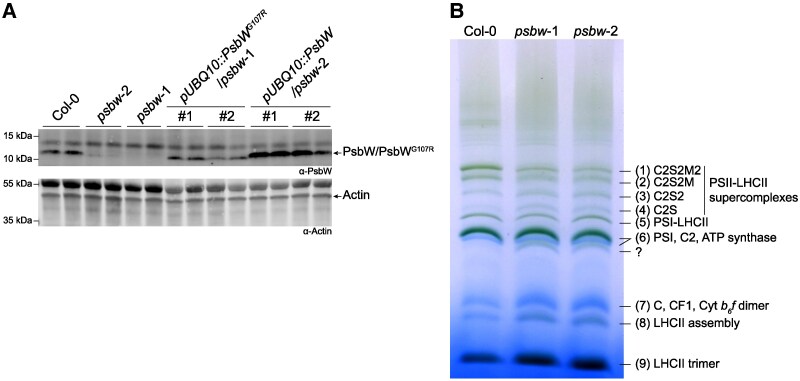
Immunoblot analysis and thylakoid complex phenotypes of Arabidopsis *psbw* mutants and wild type (Col-0). The *psbw*-2 (line #144) plants were analyzed in the M_4_ generation. **A)** Immunoblot of total leaf protein extracts using antibodies against PsbW (top panel) and actin (lower panel) as loading control. Gels were loaded based on an equal leaf area basis; two biological replicates are shown for each line. The #1 and #2 lines of the complementation lines indicate two independent T_3_ generation lines. **B)** Blue-native PAGE analysis of thylakoid membrane protein complexes, solubilized in the presence of 1% of digitonin. The observed complexes are: (1–4) PSII-LHCII supercomplexes; (5) PSI-LHCII complex; (6) PSI complexes, PSII-core dimers (C2), and ATP synthase; (7) PSII-core monomer (C), coupling factor 1 (CF1), and cytochrome (Cyt) b6f dimer; (8) LHCII assembly; and (9) LHCII trimer. The question mark indicates an unknown complex present in the *psbw* mutants.

Immunoblot analysis showed that the mutated PsbW^G107R^ protein is still present in *psbw*-2 but at greatly reduced levels compared to the wild type (Fig. 2A). No PsbW could be detected in the previously described *psbw*-1 knockout mutant, which carries a T-DNA insertion in intron 1 of the *PsbW* gene (García-Cerdán et al. 2011). Surprisingly, however, the *psbw*-1 mutant had wild-type-like starch granules (Fig. 1A). To test whether the PsbW^G107R^ mutation indeed causes the altered granule morphology of *psbw*-2, we overexpressed the wild-type version of the PsbW protein (*pUBQ10::PsbW*) in the *psbw*-2 background. Single-insert lines were selected in the T_2_ generation (based on a 3:1 ratio of hygromycin resistant to nonresistant seedlings), and homozygous T_3_ lines were selected. The presence of increased amounts of the PsbW protein was confirmed by immunoblotting (Fig. 2A). This construct complemented the mutant, restoring the granule phenotype to that of the wild type (Fig. 1A). We also expressed the mutated version PsbW^G107R^ (*pUBQ10::PsbW^G107R^*) in the *psbw*-1 knockout and selected homozygous single-insert lines following the same procedure (Fig. 2A). Interestingly, these lines exhibited the granule phenotype of the *psbw*-2 mutant (Fig. 1A). These data confirm that the PsbW^G107R^ mutation causes the starch granule abnormality in *psbw*-2 and indicate that the residual PsbW^G107R^ protein present in *psbw*-2 is neomorphic, having an altered function.

### The single amino-acid change in PsbW^G107R^ affects photosystem ii complex assembly

PsbW is a thylakoid-localized protein proposed to function in assembly and stabilization of supercomplexes formed between photosystem II (PSII) and light-harvesting complex II (LHCII) (Shi et al. 2000; García-Cerdán et al. 2011). We re-evaluated the formation of complexes between these thylakoid proteins in the *psbw* mutants using blue-native PAGE (Fig. 2B). This method separates protein complexes corresponding to PSII-LHCII supercomplexes of different antenna sizes (with C, S, and M representing the PSII core, strongly bound and moderately bound LHCII trimers, respectively), among other complexes (Sárvári et al. 2022). We observed a reduction in the largest PSII-LHCII supercomplexes (C2S2M2 and C2S2M) in both *psbw* mutants, consistent with previous reports on *psbw*-1 (García-Cerdán et al. 2011). As might be expected, with the reduction of C2S2M2 and C2S2M, we observed an increased abundance of complexes corresponding to the smaller PSII-LHCII supercomplexes C2S2 and C2S, the LHCII assembly, and LHCII trimer. The 2 bands containing, amongst others, the PSII dimer C2 and PSII core monomer (C) also showed an increased intensity. In addition, we detected an extra band in both *psbw* mutants. The identity of this band is unknown, but the complex likely contains LHCII, as it is green. These results support the hypothesis that PsbW stabilizes photosynthetic supercomplexes. Despite the detection of PsbW^G107R^ in the immunoblot analysis, the similarity of the thylakoid protein complex profile in both *psbw* mutants indicates that the residual PsbW^G107R^ in *psbw*-2 cannot fulfill its original protein function.

### PsbW^G107R^ is still membrane associated and has a dosage-dependent effect

**Figure 3.**
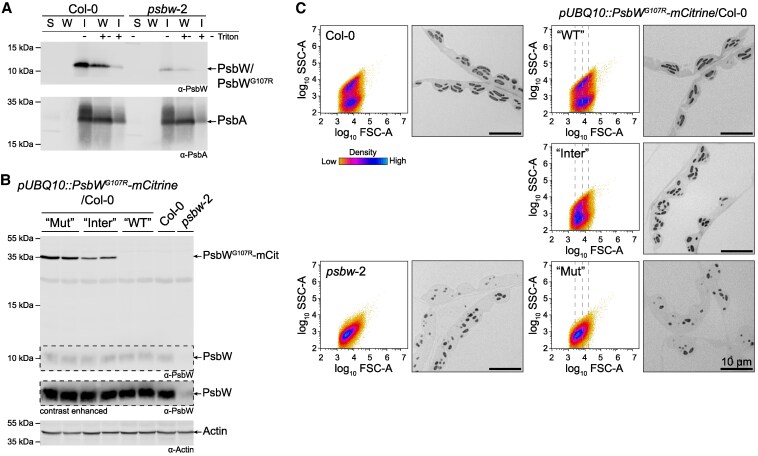
PsbW^G107R^ remains membrane associated and has a dosage-dependent effect on granule morphology. **A)** Immunoblot comparing the membrane association of PsbW and PsbW^G107R^ in *psbw*-2 and Col-0 wild type. Proteins were extracted in native buffer without detergent. After centrifugation, the membrane-containing pellet was washed either in the same buffer or in one containing 1% (v/v) Triton-X100. Depicted are the soluble (S, prior to wash), wash (W) and insoluble (I, after wash) protein fractions treated with or without Triton-X100. Membrane-bound protein PsbA served as control. **B)** Immunoblot comparing the protein amount of PsbW^G107R^-mCitrine in multiple T_2_ plants derived from the same *pUBQ10::PsbW^G107R^-mCitrine*/Col-0 T_1_ plant, using the antibodies against PsbW (upper panel) and actin (loading control). A small amount of PsbW^G107R^ is still detected in *psbw*-2, which is used as a control. For each phenotype two biological replicates are shown. “Mut”, mutant-like; “Inter”, intermediate; “WT”, WT-like. **C)** Visualization of starch granule morphology of the same T_2_ plants as in B. Left panels: Two-dimensional scatter plot of forward scatter (FSC-A) against sideward scatter (SSC-A) of purified starch granules from leaf tissue harvested at the end of night (one representative biological replicate shown; 100,000 particles measured). The dashed lines indicate the beginning and end of the density center in the mutant-like plants (“Mut”). Right panel: Light micrographs of chemically fixed and embedded leaf sections harvested at the end of the day. Scale bar unit is shown in the last image and applies to all light micrographs.

Next, we analyzed whether the change from a non-polar, neutral glycine within the transmembrane domain to an arginine, a hydrophilic amino acid, affected integration of the protein PsbW^G107R^ into the thylakoid membrane. We performed immunoblot analysis of water-soluble and water-insoluble (i.e. membrane-containing) protein fractions from the wild type, *psbw*-2, and the lines overexpressing either PsbW proteoform (*pUBQ10::PsbW/psbw*-2 and *pUBQ10::PsbW^G107R^/psbw*-1). PsbW was detected in the insoluble, but not the soluble fractions of both wild-type (Fig. 3A) and *pUBQ10::PsbW/psbw*-2 (Supplementary Fig. S2) plants. However, a considerable fraction could be solubilized when washing the insoluble pellet with the detergent Triton X100. This partitioning behavior was similar for PsbW^G107R^ in *psbw*-2 (Fig. 3A) and *pUBQ10::PsbW^G107R^/psbw*-1 (Supplementary Fig. S2) plants. The membrane protein PsbA, which forms the PSII reaction center and has multiple transmembrane helices (Trebst 1986), showed similar Triton-dependent solubilization. These data confirm the membrane association of PsbW and show that it is not affected by the G107R substitution.

Although PsbW^G107R^ remains expressed and membrane bound in the *psbw*-2 mutant, the segregation ratio of the mutant phenotype in the backcrossed F_2_ generation indicated a monogenic recessive mutation rather than a dominant or semi-dominant one. Additionally, the mutant phenotype is complemented by introducing wild-type PsbW (*pUBQ10::PsbW/psbw*-2; Fig. 1A). We reasoned that the phenotype may be influenced by the ratio of mutated to wild-type PsbW, but due to similar molecular weight, we were unable to discriminate between the two proteoforms by immunoblotting. We therefore introduced PsbW^G107R^ tagged with a C-terminal mCitrine (*pUBQ10::PsbW^G107R^-mCitrine*) into the wild type and selected a single-insertion line in the T_2_ generation. When grown on non-selective medium, this segregating T_2_ population gave rise to plants with undetectable, intermediate, or high levels of PsbW^G107R^-mCitrine (Fig. 3B), likely reflecting wild-type, hemizygous, and homozygous segregants, respectively. The levels of endogenous, wild-type PsbW were similar to Col-0 in all T_2_ plants (Fig. 3B). Notably, the abundance of PsbW^G107R^-mCitrine corresponded with the severity of granule abnormality (Fig. 3C). Plants without PsbW^G107R^-mCitrine had wild-type-like granules, while plants with high levels of PsbW^G107R^-mCitrine had granules like *psbw*-2. Plants with intermediate levels of PsbW^G107R^-mCitrine had an intermediate phenotype; their starch granules were sized between those of the wild type and *psbw*-2, with a wide range of SSC-A values. This intermediate phenotype was also observable by light microscopy (LM), where the starch granules were morphologically similar to wild-type granules, but smaller, more numerous, and less neatly arranged, as in the *psbw*-2 mutant. These data show that the granule abnormality increases with PsbW^G107R^ abundance and that the mCitrine tag does not affect the ability of PsbW^G107R^ to cause this phenotype. We also observed that F_1_ plants from a cross between *psbw*-2 and *psbw*-1, which have only one copy of the *psbw*-2 allele, do not show the mutant phenotype (Supplementary Fig. S3A). The level of the PsbW^G107R^ protein in these F_1_ plants was very low, and it was necessary to load much more protein extract to obtain any signal by immunoblot analysis (Supplementary Fig. S3B).

This dosage-dependent effect of PsbW^G107R^ appeared to be influenced by the presence of wild-type PsbW. Higher amounts of PsbW^G107R^-mCitrine were required to induce granule abnormality in the wild-type background compared with the amount of PsbW^G107R^ protein in the *psbw*-2 mutant itself (Fig. 3B). These results, together with the fact that it was possible to complement the *psbw*-2 phenotype by introducing wild-type PsbW, suggest that the effect of PsbW^G107R^ is diminished by the presence of wild-type PsbW. Potentially the two proteoforms may compete for insertion into the thylakoid membrane or, once in the membrane, for binding sites to other associated proteins.

### PsbW^G107R^ causes disordered thylakoid membrane organization

**Figure 4.**
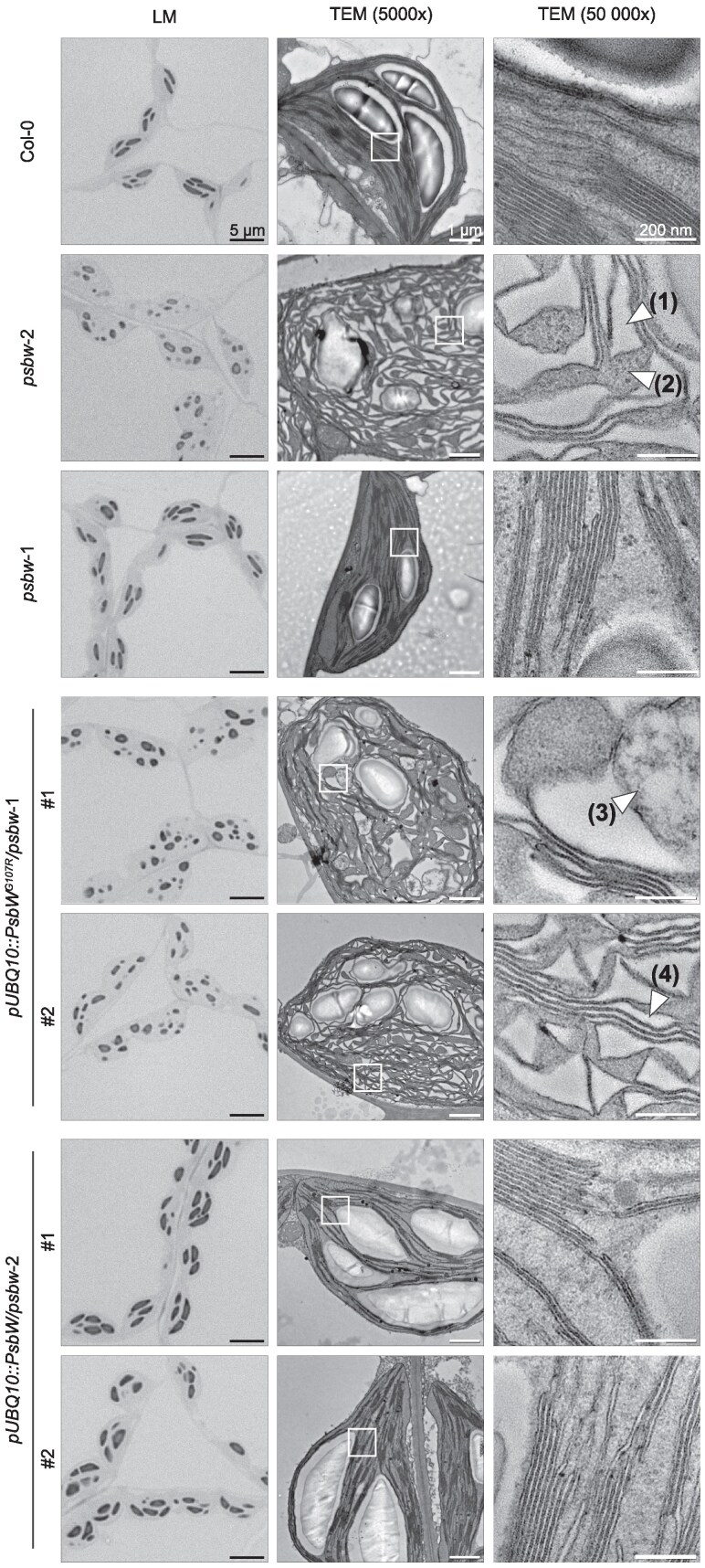
PsbW^G107R^ expression leads to disorganization of thylakoid membranes. Left panels: Light micrographs of chemically fixed and embedded leaf sections of the indicated genotypes harvested at the end of day. Middle and right panels: Transmission electron micrographs of the same embedded leaf sections as on the left, using two different magnifications. White boxes indicate the part enlarged in righthand panels. Numbered arrows indicate (1) luminal space, (2) stromal space, (3) possible mix of lumen and stroma, and (4) irregular luminal space between appressed membranes. Scale bar units are shown in the first image of each column and apply to all images in that column.

Light microscopy of *psbw*-2 leaf sections revealed many abnormally rounded chloroplasts (Fig. 3C), prompting us to examine the chloroplast ultrastructure using transmission electron microscopy (TEM). The thylakoid membranes in *psbw*-2 appeared highly disordered and poorly stacked (Fig. 4). In addition, the volume of the thylakoid lumen was greatly enlarged and, even when membrane stacks were present, the distance across the luminal space was irregular. Interestingly, higher PsbW^G107R^ levels caused a more severe phenotype; in line #1 of *pUBQ10::PsbW^G107R^/psbw*-1 (which expresses the most PsbW^G107R^, Fig. 2A), the thylakoid membrane continuity seemed to be disrupted, and in some areas, the lumen and stroma even appeared to merge. In contrast, the chloroplast ultrastructure appeared normal in the *psbw*-1 mutant, which lacks the PsbW protein altogether. It was also wild-type-like in the *pUBQ10::PsbW/psbw*-2 complementation lines, where overexpression of the wild-type PsbW rescued the abnormal starch granule phenotype. To ensure that the observed thylakoid deformations were not caused by potential artifacts of the chemical fixation procedure, we confirmed the phenotype using TEM of samples prepared with high-pressure freezing and freeze substitution for sample dehydration (Otegui 2021), and SEM of isolated thylakoids sectioned by focused ion beam milling (Supplementary Fig. S4).

**Figure 5.**
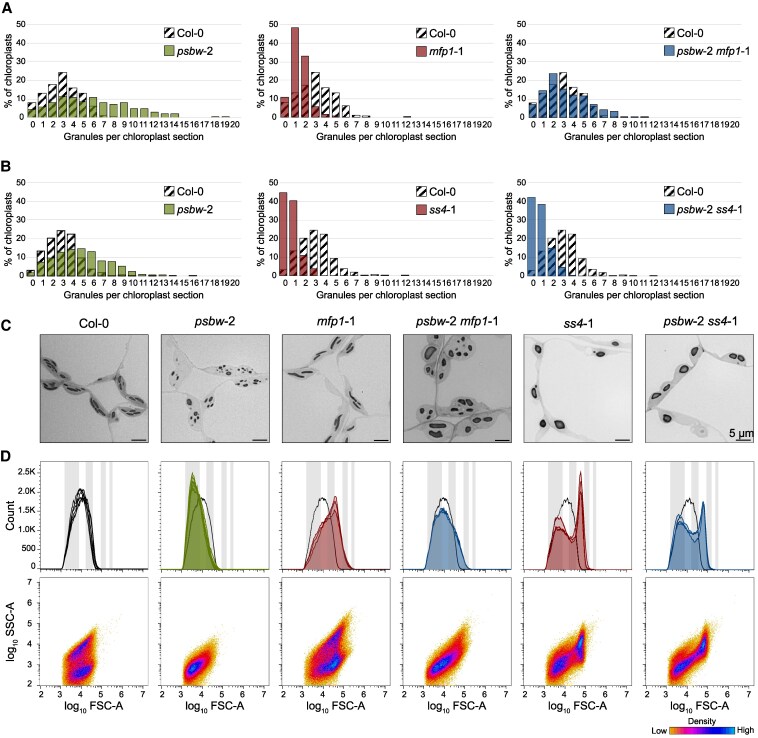
Number and morphology of starch granules in the double mutants *psbw*-2 *mfp1*-1 and *psbw*-2 *ss4*-1. **A)** Histograms of starch granule count per chloroplast section (number of chloroplasts assessed: Col-0 wild type, 343; *psbw*-2, 255; *mfp1*-1, 337; *psbw*-2 *mfp1*-1, 256). The data for the wild type (Col-0; *crosshatched bars*) is the same in all comparisons. The data shown derived from one leaf section per line; second biological replicates gave similar results (Supplementary Fig. S5A). **B)** Histograms of starch granule count per chloroplast section (number of chloroplasts assessed: Col-0 wild type, 598; *psbw*-2, 566; *ss4*-1, 296; *psbw*-2 *ss4*-1, 467). The data for the wild type (Col-0; *crosshatched bars*) are the same in all comparisons. The data shown derived from one leaf section per line; second biological replicates gave similar results (Supplementary Fig. S5B). **C)** Light micrographs of chemically fixed and embedded leaf sections of the same plants as in A/B. Scale bar unit is shown in the last image of the row and applies to all images in that row. **D)** Flow cytometry analysis of starch granule morphology of leaf starch collected at the end of day, presented as described for Fig. 1 (*n* = 3 plants; 100,000 particles measured per biological replicate). Plants of *mfp1*-1 and *psbw*-2 *mfp1*-1, as well as *ss4*-1 and *psbw*-2 *ss4*-1, were grown in two separate batches, each alongside Col-0 and *psbw*-2 as controls. Therefore, for Col-0 and *psbw*-2, graphs of 6 plants are shown. The lower panels (SSC-A vs. FSC-A) indicate granule shape of a representative sample; data were consistent across the biological replicates.

The formation of starch granules in the stromal pockets between thylakoid membranes is facilitated by MFP1, which directs the starch initiation proteins to specific stromal sites adjacent to the thylakoid membrane (Sharma et al. 2024). Since MFP1 itself is anchored to the thylakoids via a transmembrane domain, we tested whether the starch granule phenotype is caused by an effect of the disordered thylakoid membrane system on MFP1. Therefore, we created the *psbw*-2 *mfp1*-1 double mutant and assessed its starch granule phenotype. As observed previously, the *mfp1*-1 mutant had impaired granule initiation, with most chloroplast sections containing far fewer granules than the wild type (Fig. 5A and C; Seung et al. 2018). These granules grew larger than in the wild type but remained lenticular in shape (Fig. 5C and D). In contrast, *psbw*-2 again displayed a highly disrupted membrane system and more, small, irregularly shaped granules per chloroplast section. Comparing the double mutant phenotype with the parental phenotypes, we observed an additive phenotype (Fig. 5). On one hand, the double mutant had fewer, larger granules compared to *psbw*-2, indicating that the absence of MFP1 lowers the number of initiation events in the *psbw*-2 background. On the other hand, the granules were still irregularly and abnormally shaped, and still more abundant than in the wild type. These data show that MFP1 is still functional in *psbw*-2, but that the starch phenotype of *psbw*-2 is unlikely to be caused solely by an effect of PsbW^G107R^ on MFP1. Rather, the starch phenotype is likely due, at least in part, to the disrupted thylakoid membrane organization caused by PsbW^G107R^.

While MFP1 plays a crucial role in localizing starch granule initiation, SS4 is known to be a central player in initiating granule formation itself. Therefore, we also created the *psbw*-2 *ss4*-1 double mutant. The *ss4*-1 single mutant showed the characteristic drastic reduction in granule numbers per chloroplast section, with most chloroplast containing 0 or 1 large granule (Fig. 5B and C). As well as being a crucial factor for granule initiation, SS4 also plays a role in determining granule shape. In the absence of SS4, the granules grow isotropically and become more spherical (Fig. 5C), which is also evident in the FC analysis, where the scatter plot (FSC-A vs. SSC-A) shows only one density center (Fig. 5D). In the *psbw*-2 *ss4*-1 double mutant, the *ss4*-1 mutation has a dominant effect over the *psbw*-2 mutation, regarding granule number and shape (Fig. 5B and C). While the membrane system is still highly disrupted like in *psbw*-2 (Supplementary Fig. S5C), most chloroplast sections show 0 or only 1 large, round granule like in *ss4*-1. These data suggest that the granules in *psbw*-2 are still initiated through the canonical initiation process depending on SS4. They also suggest that SS4 regulates granule morphology also in the disordered membrane system of *psbw*-2.

### Photosynthesis and growth are affected by overexpression of mutated PsbW^G107R^ protein

**Figure 6.**
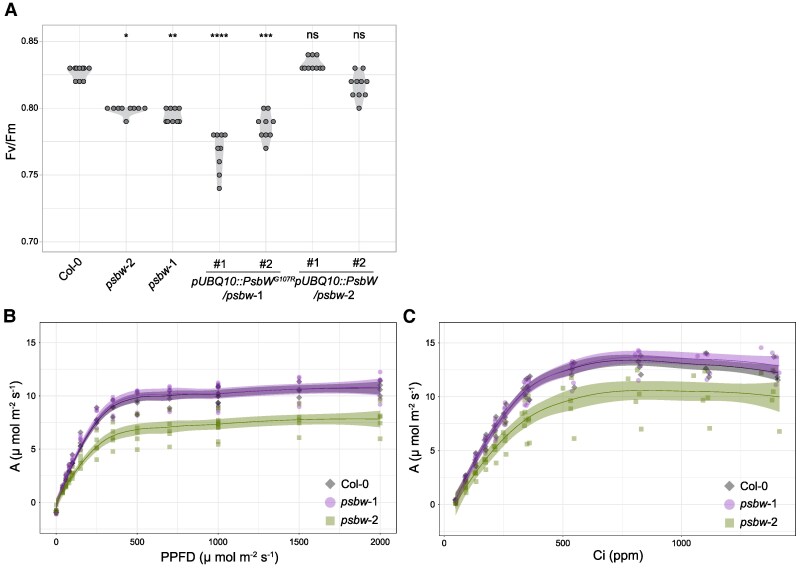
Maximum quantum yield of PSII, light response curve and CO_2_ response curve of *psbw* mutants compared to the Col-0 wild type. **A)** Maximal quantum yield of PSII (F_v_/F_m_) (*n* = 9 to 10 plants). The statistical significance of differences to Col-0 was analyzed using the Kruskal–Wallis test followed by Dunn's multiple comparison test. ns, not significant; *P < 0.05; **P < 0.01; ***P < 0.001; ****P < 0.0001. **B)** Light response curve measured on 5- to 6-week-old plants (*n* = 4 to 5). **C)** CO_2_ response curve measured on 5- to 6-week-old plants (*n* = 4 to 5). Dots show individual data points. Light shades around curves in **B** and **C** denote SE. The values of Col-0 and *psbw*-1 strongly overlap.

To address whether the disordered thylakoid arrangement of plants expressing PsbW^G107R^ affects the photosynthetic light reactions, we investigated their photosynthetic performance by analyzing the maximum quantum yield of PSII (F_v_/F_m_) using pulse amplitude modulated (PAM) fluorometry. Dark-adapted wild-type plants had an F_v_/F_m_ of 0.83 ± 0.000 (SE), a value typical for most vascular plant species under standard growth conditions (Fig. 6A; Björkman and Demmig 1987). The *psbw*-1 mutant showed a small but significant reduction in F_v_/F_m_ (0.79 ± 0.001), as previously reported (García-Cerdán et al. 2011), showing that the absence of wild-type PsbW slightly impairs PSII function. A similar decrease in F_v_/F_m_ was also observed in *psbw*-2 (0.80 ± 0.000) and was even more pronounced in the *pUBQ::PsbW^G107R^/psbw*-1 lines. This was particularly evident in line #1 (0.77 ± 0.002), which showed the highest PsbW^G107R^ expression (Fig. 2A) and also the most severe alteration in thylakoid arrangement (Fig. 4). These data suggest that high levels of PsbW^G107R^ reduce photosynthetic efficiency more than the simple loss of PsbW, likely due to the disrupted membrane continuity, as observed in the TEM images (Fig. 4). In contrast, the F_v_/F_m_ values of the complementation lines *pUBQ::PsbW/psbw*-2 were restored to wild-type values (0.83 ± 0.000 and 0.82 ± 0.001).

To further investigate whether *psbw-*1 and *psbw*-2 exhibited altered phenotypes in the Calvin-Benson–sBassham cycle or the electron transport chain, the leaf gas exchange dynamics were measured with an infrared gas analyzer equipped with a PAM fluorometer. Despite differences in instrument settings in this compared with the previous experimental setup (e.g. modulation frequency and pattern), the reduced F_v_/F_m_ values for *psbw*-1 and *psbw*-2 plants (0.77 ± 0.004, and 0.77 ± 0.002, respectively) relative to the wild type (0.81 ± 0.005) was consistent.

Next, we determined the saturating light intensity for CO_2_ assimilation. Light response (A/Q) curves measured from 0 to 2000 µmol photons m⁻² s⁻¹ revealed that net CO₂ assimilation rate (A) plateaued at 500 µmol photons m⁻² s⁻¹ for all 3 genotypes (Fig. 6B). The light-saturated values for A were similar for wild-type and *psbw*-1 plants, but lower for *psbw*-2, indicating an impaired CO₂ assimilation capacity in this line under high light conditions. The intercellular CO₂ concentration response (A/C_i_) curves were subsequently measured (at 1000 µmol photons m⁻² s⁻¹ to ensure that saturating A was reached). As shown in Fig. 6C, the *psbw*-2 mutant exhibited a CO₂ assimilation penalty under high light and high CO_2_ conditions, similar to the observations from the A/Q curves.

The A/Ci curves were used to derive biologically meaningful parameters using the extended FvCB model (Farquhar et al. 1980; Yin et al. 2021)—specifically, the CO_2_-saturated maximum carboxylation rate of Rubisco (V_cmax_), light-saturated potential electron transport rate (J_max_), and triose phosphate utilization (TPU). The *psbw*-2 mutant showed a significant reduction in V_cmax_ (*P* = 0.02749) and a substantial reduction in J_max_ compared with wild-type plants (the latter of borderline statistical significance; *P* = 0.05004; Table 1), indicating an impairment in CO_2_ utilization and electron transport efficiency. The TPU describes the decline in CO₂ assimilation rate (A) with increasing CO₂ concentrations in high light conditions in the A/Ci curves, which underlies the limited capacity to synthesize sugars from triose phosphates (Yang et al. 2016). However, no statistical differences between the TPU values of wild type and mutants were detected (*p^psbw^*^1^ = 0.77283, *p^psbw^*^-2^ = 0.14164). Furthermore, we evaluated non-photochemical quenching (NPQ) performance in a stepwise transition to highlight (Supplementary Fig. S6), but no statistical differences were observed.

**Table 1.** Fitted parameters from the extended FvCb model of PsbW mutants compared with the wild type (Col-0)

Genotype	V_cmax_	J_max_	TPU
wild type (*n* = 4)	43.81 ± 1.57	80.46 ± 2.68	4.45 ± 0.14
*psbw*-1 (*n* = 5)	43.85 ± 2.05 ns	81.52 ± 3.36 ns	4.56 ± 0.22 ns
*psbw*-2 (*n* = 5)	33.42 ± 2.91*	63.35 ± 6.53 ns	3.68 ± 0.36 ns

V_cmax_, CO_2_-saturated maximum carboxylation rate of Rubisco (µmol CO_2_ m⁻² s⁻¹); J_max_, light-saturated potential electron transport rate (µmol e- m⁻² s⁻¹); and TPU, triose phosphate utilization (µmol CO_2_ m⁻² s⁻¹). ±denote SE. The statistical significance of differences to Col-0 was analyzed using the Kruskal–Wallis test followed by Dunn's multiple comparison test. ns, not significant; *P < 0.05, **P < 0.01.

**Figure 7.**
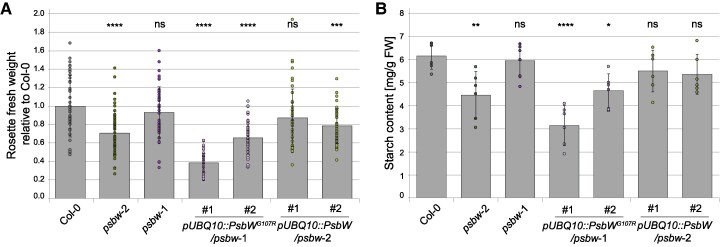
Fresh weight and starch content measurements of *psbw* mutants and complementation lines compared to the Col-0 wild type. **A)** Fresh weight of 35-day-old rosettes from 3 independent experiments, normalized to the respective Col-0 wild type (*N* = 3; *n* = 13–16 rosettes). **B)** Leaf starch content at the end of day (*n* = 6 rosettes). Dots show individual data points. Error bars denote SD. Statistical significances of differences to Col-0 were tested by performing one-way ANOVA and Dunnett's multiple comparison test. ns, not significant; * *P* < 0.05; **P < 0.01; ***P < 0.001; ****P < 0.0001.

Consistent with the reduced V_cmax_ and J_max_ of *psbw*-2, we observed a slight decrease in growth (Supplementary Fig. S7) and a reduced rosette fresh weight of 35-day-old *psbw*-2 mutant plants (Fig. 7A) under standard growth conditions. The overexpression line #1 of *pUBQ::PsbW^G107R^/psbw*-1, which was also most affected in the F_v_/F_m_ and thylakoid organization, showed the lowest amount of fresh weight. Only line #1 of the complementation lines (*pUBQ::PsbW/psbw*-2) had fully restored growth, while line #2 still showed a significant reduction in rosette fresh weight. We also analyzed the starch content of these plant lines to assess whether the decrease in photosynthetic capacity and the altered granule phenotype affected the total amount of starch (Fig. 7B). The *psbw-*2 mutant and both lines #1 and #2 of *pUBQ::PsbW^G107R^/psbw*-1 had lower starch contents than the wild type, with overexpressing line #1 having the least starch.

## Discussion

In this study, while searching for mutants with abnormal starch granules, we identified the *psbw*-2 mutant, which affects a small PSII-associated integral membrane protein, PsbW. The resulting glycine-to-arginine conversion within the membrane-spanning domain of PsbW is neomorphic and causes extreme distortion of the thylakoid membrane system—something not seen upon complete loss of PsbW. We first discuss the impact of the membrane distortion on starch granule biogenesis, before considering the special features of the PsbW^G107R^ proteoform, its impact on the thylakoids, and the consequences for photosynthesis.

### Thylakoid membrane disorder impacts granule number and shape

The starch granule phenotype of Arabidopsis plants expressing PsbW^G107R^ is strikingly different from that of the wild type and any other known mutant affected in starch metabolism or PSII organization. While some photosynthesis-related mutants exhibit reduced starch content, their impact on starch granule number and morphology has rarely been examined. In *psbw*-2, the granules were considerably smaller, irregularly shaped, and far more numerous than in the wild type (Fig. 1A; Fig. 5A). The increase in granule number per chloroplast section was not due to an increased rate of starch biosynthesis, since *psbw*-2 had less total starch than the wild type (Fig. 7B). This decrease in starch content is likely due to the reduction in carbon fixation capacity (V_cmax_) (Fig. 6 and Table 1). Alternatively, the starch turnover—the simultaneous breakdown during net assimilation (Stitt and Zeeman 2012)—could be affected by the increased surface area to volume ratio in *psbw*-2 (Roldán et al. 2007; Scialdone et al. 2013). The higher granule number is likely a consequence of its altered thylakoid architecture. Electron microscopy suggested that this altered thylakoid arrangement results in smaller and more numerous stromal pockets—a finding verified with 3 different microscopy methods (Fig. 4; Supplementary Fig. S4). Recent advanced microscopy and isotope labelling analyses showed that multiple starch granule initials are generated within the same stromal pocket and can fuse within the first hour of synthesis (Bürgy et al. 2021). It thus seems plausible that, in *psbw-*2, starch granule initiation occurs normally, but that these events are distributed between an increased number of smaller stromal pockets, ultimately leading to less fusions and an increased number of granules per chloroplast (Fig. 5A).

In Arabidopsis, the granule initiation pathway centers around SS4, which is proposed to elongate glucan primers delivered by PTST2 (Seung et al. 2017). PTST2 associates with the membrane-anchored MFP1 and both proteins co-localize into multiple puncta—presumably defining the sites of granule initiation (Seung et al. 2018, 2017; Sharma et al. 2024). The proper location of PTST2 with MFP1 is important for the initiation of the correct number of granules (Seung et al. 2018; Sharma et al. 2024). In the absence of MFP1, YFP-tagged PTST2 still accumulated in puncta, albeit only one or two; presumably, these are the sites where the reduced number of granules is initiated in the *mfp1* mutant (Seung et al. 2018). Since the loss of MFP1 in the *psbw*-2 background reduced the starch granule numbers per chloroplast, MFP1 must, to some extent, be functional within the altered thylakoid system (Fig. 5). One possibility is that the distribution of MFP1 into multiple puncta on the thylakoid membrane is altered in the *psbw*-2 mutant. Such an effect was recently investigated in another Arabidopsis mutant with an altered starch granule morphology—in this case caused by the loss of FZL, a membrane-associated protein proposed to regulate the organization of the thylakoid membrane network (Liang et al. 2018). However, the punctate localization of MFP1 was retained, and it was concluded that the redistribution of MFP1 was not the primary cause of that phenotype (Esch et al. 2022). Congruent with this, our analysis of the *psbw*-2 *mfp1*-1 double mutant also showed that the granule count—while reduced relative to *psbw*-2, was still considerably higher than in the *mfp1*-1 single mutant. Thus, the irregular thylakoids and associated increase in stromal pockets seem to separate the granule initiation events even when not thylakoid anchored via MFP1. These initiation events may still be promoted by stromal SS4 and PTST2, which is supported by the observation that in the *psbw*-2 *ss4*-1 double mutant we observed an *ss4*-1-like number of granules per chloroplast section (Fig. 5B).

The deformed stromal pockets could also explain the irregular starch granule shape in *psbw*-2. SS4 is proposed to have an important role in concentrating the activities of starch biosynthesis proteins in the equatorial region of the developing wild-type granules, leading to an anisotropic deposition of newly synthesized glucan and their characteristic lenticular shape (Bürgy et al. 2021). Indeed, the few granules produced in the *ss4* mutant grow isotropically and are near-spherical. Intriguingly, the N-terminal region of SS4, which contains putative protein-protein interaction motifs, is essential both for the correct localization of SS4 and for its role in controlling the pattern of granule growth (Gámez-Arjona et al. 2014; Raynaud et al. 2016; Lu et al. 2018). Although the exact mechanism is not understood, it is plausible that the altered stromal pocket shape in *psbw*-2 may influence the localization of SS4 and thereby alter the growth direction. This is consistent with the observation that, despite the disrupted thylakoid organization, the granules in the *psbw*-2 *ss4*-1 double mutant exhibit a nearly spherical morphology (Fig. 5C and D, Supplementary Fig. S5C). This is also consistent with conclusions drawn from studying the Arabidopsis *fzl* mutant, which produces starch granules with an irregular surface matching the irregular outlines of its stromal pockets (Esch et al. 2022).

It is worth noting that the influence of plastid membranes on starch formation may not be limited to chloroplasts. Young amyloplasts from barley and maize contain internal membranes in the vicinity of the starch grains, which have been hypothesized to form pockets influencing granule initiation and starch deposition (Buttrose 1960; Shannon et al. 2009). In-line with this, the hypomorphic *opaque5* maize mutant (impaired in monogalactosyldiacylglycerol synthase MDG1), which has a reduction in the total galactolipid abundance of its amyloplast membranes, produces membrane-separated compound granules derived from multiple initiation events instead of simple granules (Myers et al. 2011). Thus, we suggest that the plastid endomembrane system and its role in the synthesis of both storage and transient starch merits further study.

### The glycine-to-arginine substitution in PsbW leads to a drastic membrane phenotype

Our results suggest that the aberrant membrane architecture in *psbw*-2 is caused by the accumulation of the mutant proteoform, the G107R substitution of which lies in its transmembrane α-helix. Arginine is positively charged in nearly all environments, is highly hydrophilic (Kyte and Doolittle 1982; Biswas et al. 2003) and is rarely found in the hydrophobic hydrocarbon core of the lipid bilayer (Ulmschneider et al. 2005; Hristova and Wimley 2011). The low abundance of the PsbW^G107R^ protein in *psbw*-2 could mean that it is less stable than the wild-type protein. Nonetheless, the residual PsbW^G107R^ still appears to integrate into the membrane, judging from its partitioning in solubility assays (Fig. 3A). This is not without precedent: in some ion channels, one membrane-spanning α-helix (S4) contains 4 arginines that are critical in voltage-sensing. However, its membrane insertion entails the penetration of water molecules into the bilayer and slight membrane thinning, allowing the arginine residues to form an extensive network of hydrogen bonds with water and the phosphate groups of lipids (Krepkiy et al. 2009). Although PsbW^G107R^ carries only a single arginine, it is located close to the helix center, where its impact may be greatest (Hristova and Wimley 2011). Thus, PsbW^G107R^ may alter the structure of the thylakoid membranes in its immediate vicinity. This impact may also be particularly evident due to the unique lipid composition of the thylakoids, which consist predominantly of uncharged glycolipids monogalactosyldiacylglycerol (MGDG) and digalactosyldiacylglycerol (DGDG). Anionic sulfo- and phospholipids each constitute only ∼10 mol% (Kobayashi et al. 2017). This unusual composition, together with certain membrane proteins, allows for membrane flexibility required for the extreme curvature of the membrane at the grana margins (Armbruster et al. 2013; Jouhet 2013). The introduction of a charged arginine and the resulting water penetration might lead to more severe deformations in the flexible thylakoid membranes compared to phospholipid-dominated membranes. If membrane thinning and water penetration indeed occur, this may to some extent permeabilize the thylakoids and contribute to the observed luminal swelling and reduced photosynthetic efficiency (Figs. 4 and 6).

Given the observed dosage effect of PsbW^G107R^ (Fig. 3), it appears that the more PsbW^G107R^ is expressed and inserted into the membrane, the greater the membrane deformation. These effects may depend on the exact localization of PsbW^G107R^ and be more pronounced where multiple PsbW^G107R^ molecules are inserted in close proximity. Interestingly, however, our data also suggests that the effect of PsbW^G107R^ is influenced by the presence or absence of the wild-type PsbW protein. Firstly, the expression of the wild-type PsbW protein can complement the *psbw*-2 mutant phenotype (Fig. 1A). Secondly, in lines expressing mCitrine-tagged PsbW^G107R^ in the wild-type background, the severity of the mutant phenotype depended on the ratio between mutant and wild-type PsbW (Fig. 3B and C). This suggests that the effects of the PsbW^G107^ are to some extent conditional, perhaps on it positioning at specific sites in the thylakoids, for which it has to compete with the wild-type PsbW protein. Alternatively, both proteins could be competing for insertion into the thylakoids, with the wild-type protein preventing insertion or displacing the mutant version.

### PsbW^G107R^ likely impedes PSII supercomplex formation via multiple factors

The reduction in PSII supercomplex formation is seen both in *psbw*-1, which lacks PsbW altogether, and *psbw*-2, which contains reduced levels of PsbW^G107R^ protein (Fig. 2). Yet, it is unlikely that the reduction in PSII supercomplexes in *psbw*-2 is merely caused by reduced PsbW^G107R^ protein levels, since a ∼75% decrease in PsbW protein was previously associated with only a mild decrease in PSII supercomplexes (García-Cerdán et al. 2011). The model of the Arabidopsis PSII supercomplex based on cryo-transmission electron microscopy and single-particle analysis (Su et al. 2017; Van Bezouwen et al. 2017; Sheng et al. 2018) does not indicate any interactions between G107 of PsbW and other proteins. However, we propose that its substitution with arginine could well impede PSII supercomplex formation due to changes in the positioning of its N- and C-terminal ends. In the case of the human FIBROBLAST GROWTH FACTOR RECEPTOR 3 (FGFR3), a comparable glycine-to-arginine conversion in its single α-helical transmembrane domain not only caused membrane thinning but also altered the positioning of the protein within the membrane, such that the arginine was closer to the bilayer interface. This pulled hydrophilic residues at the other end of the transmembrane helix into the bilayer periphery (Han et al. 2006). If the membrane position of PsbW^G107R^ was shifted in a similar manner, this would result in a longer N-terminal luminal extension, while part of its C-terminal stromal tail would be pulled into the membrane (Fig. 1). Both termini of PsbW were hypothesized to contribute to protein-protein interactions (García-Cerdán et al. 2011; Van Bezouwen et al. 2017), and the negatively charged amino acids in the N-terminal luminal region were shown to be required for the complex formation of PSII (Thidholm et al. 2002). In addition, modelling suggests that PsbW interacts with multiple glyco- and phospholipids through its luminal and stromal domains via hydrogen bonds and van der Waals forces (Sheng et al. 2018), which is important since the membrane lipid composition of PSII is essential for the formation and stabilization of the supercomplex (Kobayashi 2016). Thus, the assembly and stabilization of PSII supercomplexes likely depends on multiple structural features of PsbW that enable its interactions with proteins and lipids, which are potentially compromised in the PsbW^G107R^ proteoform.

Similar to the reduction in PSII supercomplex formation, the additional band visible in the blue-native PAGE (Fig. 2B) is unlikely to be the cause of the thylakoid membrane phenotype of *psbw*-2, as we observed it in both *psbw*-2 and *psbw*-1. However, it would be interesting to evaluate the composition of this thylakoid complex to gain a better understanding of the role of PsbW itself.

### The impact of disordered membrane organization on photosynthesis

Even though *psbw*-2 has a severe thylakoid membrane phenotype, it is surprising that photosynthesis and growth were not severely affected, at least under our standard growth conditions. Although the maximum quantum yield of PSII was reduced compared with the wild type, it was similar to that of *psbw*-1 plants (Fig. 6A). This suggests that the aberrant thylakoid system of *psbw*-2 may not be the primary cause of its lowered F_v_/F_m_ ratio, since the thylakoids of *psbw*-1 appeared normal. Rather, the lowered F_v_/F_m_ ratio may be a consequence of the defect in PSII supercomplex assembly in both cases. Plants deficient in CURVATURE THYLAKOID1 (CURT1), a protein facilitating thylakoid curvature at grana margins, also showed wild-type-like F_v_/F_m_ ratios, even though the thylakoid membranes were disorganized with extended stretches of unstacked membranes—presumably, there were still enough appressed membranes present for normal PSII function (Armbruster et al. 2013). Thus, we suggest that the thylakoids of *psbw*-2, despite being disordered, still form sufficient appressed regions for a *psbw*-1-like PSII. Only when PsbW^G107R^ is overexpressed is there a larger reduction in F_v_/F_m_. This is likely a consequence of the more severe thylakoid distortion, luminal swelling, and potential leakage of solutes across the thylakoid, which in turn may reduce photosynthetic efficiency and plant growth.

When shifting from normal growth conditions to high light and high CO₂ conditions, the phenotypic differences between wild-type, *psbw*-1, and *psbw*-2 plants became more pronounced. This was indicated by a reduction in the saturating CO₂ assimilation rates (Figs. 6B and C) and in V_cmax_ and Jₘₐₓ (Table 1). Given the similar F_v_/F_m_ values of *psbw*-1 and *psbw*-2 mutants, the penalty in V_cmax_ and Jₘₐₓ observed in *psbw*-2 is likely not due to PSII itself, but to downstream components of the electron transport chain. The membrane disorder in *psbw*-2 plants could adversely affect the photosynthetic electron transfer from PSII to PSI via plastoquinone (PQ) or plastocyanin (PC), as reflected by the lower J_max_ values. The introduction of the mutated PsbW^G107R^ into the thylakoid membrane might change the lipid and protein distribution within the thylakoids, which could impact both the lifetime and the diffusion path of the reduced PQ, potentially leading to the observed decrease in electron transfer efficiency (Kirchhoff et al. 2002). Alternatively, the increased lumen volume, as observed in TEM images (Fig. 4), could impede the diffusion of the long-range electron carrier PC, thereby disrupting the electron transport chain (Höhner et al. 2020). The disorganized thylakoids may also limit CO_2_ diffusion to the sites of carboxylation, thereby reducing mesophyll conductance and V_cmax_ (Bernacchi et al. 2002). Furthermore, since the electron transport chain and CO_2_ fixation are interconnected, the limitation of either of them will affect the other (Farquhar et al. 1980). Establishing what is the primary effect of the integration of PsbW^G107R^ into the thylakoids and what are secondary effects is well worth further investigation as it will further our understanding of how the thylakoid membrane system simultaneously enables optimal photosynthesis and coordinates the deposition of starch, one of the key photosynthetic products.

## Materials and methods

### Plant materials and growth conditions

All Arabidopsis (*Arabidopsis thaliana*) mutants were in the Columbia-0 (Col-0) background. The T-DNA insertion mutants of *PsbW* (*psbw-1*; SAIL_885_A03), *SS4* (*ss4*-1; GABI_290D11) and *MFP1* (*mfp1*-*1*; SALK_124298) genes were generated by the Syngenta Arabidopsis Insertion Library collection (Sessions et al. 2002) and Salk Institute Genomic Analysis Laboratory (Alonso et al. 2003) and acquired from The European Arabidopsis Stock Centre (NASC). The *psbw-*2 (#144) mutant was identified from the EMS-mutagenized population described below, and homozygous plants from the M_4_ generation were used for all analyses, transformations and crossings. Homozygous *psbw*-2 *mfp1*-1 and *psbw*-2 *ss4*-1 double mutants in the F_2_ generation of a cross between *mfp1*-*1* and *psbw*-2 and *ss4*-1 and *psbw*-2 were identified via PCR-based genotyping (followed by Sanger sequencing of the *psbw*-2 mutant amplicon) using the primers listed in Supplementary Table S2. Homozygous *psbw*-2 *mfp1*-1 and *psbw*-2 *ss4*-1 plants from the F_3_ generation were used for analysis. Complementation lines *pUBQ10::PsbW/psbw-2* and *pUBQ10::PsbWG107R/psbw-1* were created as described below. Unless otherwise specified, two independent T_3_ generation lines (#1, #2) from each were used for analyses. Plants were cultivated on soil in a Percival AR-95L chamber (CLF Plant Climatics), in a 12-h light/12-h dark regime at 20 °C, 150 *µ*mol photons m^−2^ s^−1^, and 65% relative humidity.

### Cloning and plant transformation

The genomic sequence of *PsbW* and *PsbW^G107R^* (including their single introns) were synthesized after the genomic DNAsequence of wild type (Col-0) and *psbw*-2, respectively, eliminating a Bpi1 restriction enzyme cutting site (synthesized by Twist Bioscience, California). They were then combined with the *pUBQ10* promotor and *tHsp* terminator with or without an in-frame *mCitrine* tag (synthesized by Twist Bioscience, CA, USA) into the pEC50505 binary vector via MoClo modular cloning (Weber et al. 2011) together with a hygromycin resistance cassette (pEC33027; *p35S::HYG:t35S*). After transformation of the binary vectors into Agrobacterium (*Agrobacterium tumefaciens*) strain GV3101 through electroporation, Arabidopsis plants were transformed using a floral dip method (Zhang et al. 2006). Transformed seedlings were selected on 0.5% (w/v) agar plates containing ½ Murashige and Skoog (MS) medium including vitamins (Duchefa Biochemie) and 25 mg/L hygromycin. The sequences of the final constructs are provided in the Supplementary Data Set 1.

### Mutagenesis and next-generation sequencing

For M_1_ seed production, 200 mg of Arabidopsis Col-0 seeds were incubated in 10 mL EMS solution (0.2% [v/v] EMS in water) on a rotating shaker at room temperature for 15 h, washed extensively with water, and transferred to soil. M_1_ plants were cultivated in a greenhouse (20 °C, 60% relative humidity, 14-h light/10-h dark regime), and M_2_ seeds collected from each individual. Leaf material from 25-day-old M_2_ plants was harvested for screening for starch granule phenotypes using FC, as described below. Selected mutants, including line #144 were further propagated via selfing for further analyses. To identify the causal mutation in line #144, an M_3_ plant was backcrossed with Col-0. Individuals exhibiting the mutant starch phenotype were identified by FC in the F_2_ generation. From these individuals, genomic DNA was extracted using the sbeadex plant DNA purification kit (LGC Genomics) and a KingFisher Flex Purification Systems (Thermo Scientific), pooled in equal amounts and subjected to whole genome sequencing. Library preparation and Illumina Novaseq 6000 sequencing were performed by the Functional Genomics Center Zurich (FGCZ) of the University of Zurich and ETH Zurich. The 150-bp paired-end reads were quality controlled, pre-processed, and aligned using the SUSHI workflow manager (Hatakeyama et al. 2016). Sequencing adapters and low-quality ends (<Q20) were removed during pre-processing of the reads. Reads that met the filtering criteria (average quality ≥ Q20, minimum length ≥18 bp) were aligned to the reference genome of Arabidopsis (TAIR10), resulting in a coverage of 70× to 130×. Frequency-based, variant calling with allele frequencies above 80% was generated using the freebayes-parallel script in freebayes (version 1.3.2 [Garrison and Marth 2012]), including only reliably mapped reads (mapping quality > Q30, base quality > Q20). Functional annotation of the variants was performed using SnpEff (version 4.3 [Cingolani et al. 2012]) against TAIR10 gene models. Sample-specific variants were identified by comparison with a Col-0 control, using SnpSift and SnpEff (version 4.3). The resulting list of single nucleotide polymorphisms (SNPs) was manually reduced to mutations that (i) could have been caused by EMS (i.e. G/C to A/T conversions), (ii) lie within a gene, and (iii)) cause nonsynonymous substitutions (altering the amino acid sequence of the encoded protein) or acceptor splice site alterations (likely leading to intron splicing defects).

### Phylogenetic sequence analyses and 3D protein model prediction

Orthologous PsbW protein sequences were identified using the blastp algorithm on the National Centre for Biotechnology Information (NCBI) website using the standard databases of nonredundant protein sequences (nr) with a E-value (expectation value) cutoff of 0.05 and the Arabidopsis PsbW protein sequence as query. Orthologs from various species per taxonomy group were selected based on a low E-values (at least 1×10^−8^) with preference for reference proteome sequences. In total, 61 PsbW proteins from 54 Viridiplantae species were used for comparative and evolutionary analyses. The full-length protein sequences were aligned using CLC Genomic Workbench 12 with default settings (alignment = very accurate, Gap open cost = 10, gap extension cost = 1) creating conservation and gap functions. WebLogo was used to create WebLogos (Crooks et al. 2004). A phylogenetic tree using the LG + G model was constructed in PhyML (Guindon et al. 2010), and support for nodes was determined by fast bootstrapping with 500 replicates. The 3-dimensional protein models were predicted by AlphaFold2 (Jumper et al. 2021) and visualized using the PyMOL Molecular Graphics System (Version 3.0 Schrödinger, LLC.).

### Starch granule purification and analysis by FC and SEM

Three medium-sized Arabidopsis leaves (50 to 100 mg, in total) from 5-week-old plants were harvested at the end of day, snap-frozen in liquid nitrogen, and homogenized for 30 s in a Retsch mixer (Retsch MM301; oscillation frequency set to 30 s^−1^, using two 4-mm glass beads per microfuge tube). The frozen powder was mixed with 900 μL starch extraction buffer (50 mm Tris-HCl, pH 8.0, 0.2 m EDTA, 0.5% [v/v] Triton X-100 and 0.8% [w/v] SDS) and centrifuged for 5 min at 4,500 *g* at 20 °C. The resulting starch-containing pellet was washed twice with water and resuspended in 500 μL water. For purification, 100 µL suspension was transferred to a 96-well filter plate with polyethylene terephthalate frit (20 *μ*m pore size; Fisher Scientific) and centrifuged for 1 min at 4,500 *g* at 20 °C. Wells were washed once with 100 μL of water to ensure starch elution. Purified starch suspensions were stored at 4 °C until use.

For FC, purified starch suspensions were diluted 1:20 in water and analyzed using a CytoFLEX (Beckman Coulter). Samples were mixed by orbital shaking (1,500 rpm for 6 s, acceleration time of 1 s) to ensure a homogeneous starch granule distribution, and 100,000 particles per sample were measured using the 488 nm laser at default power settings and a flow rate of 3,000 to 6,000 particles s^−1^. Samples with fewer than 60,000 particles were excluded. The connection between particle diameter and forward scattering (FSC) values was established as described in Thieme et al. (2023). Particles with a diameter <0.1 *µ*m (likely constituting non-starch contaminants) were excluded from the analysis, and granule distribution was visualized using FlowJo (version 10).

For SEM, purified starch samples were applied on chips sliced from Si-wafers, which then were mounted to SEM-stubs with silver glue, air dried, sputter coated with 5 nm platinum (Pt)/palladium (Pd), and imaged as described previously in (Boehlein et al. 2023).

### Fixation and embedding of leaf tissue and TEM

Leaf samples from 5-week-old plants were harvested at the end of day, chemically fixed, stained and embedded in Spurr epoxy resin (Sigma-Aldrich) as previously described in Abt et al. (2020). For LM, semi-thin sections (0.5 *µ*m) were stained with toluidine blue O and imaged using an Axio Imager Z2 microscope (Zeiss) with an Axiocam monochrome camera. TEM analysis of ultrathin (70 nm) sections from the same blocks was conducted as previously described by Liu et al. (2023).

For the high-pressure freezing and freeze substitution, small pieces were punched out of freshly harvested leaves and after degassing under vacuum in 0.1 m PBS buffer (pH 7) for 60 min they were transferred to a sandwich made of two 6 mm aluminum platelets with a sample cavity of 200 *μ*m. The sandwiches were frozen in a high-pressure freezing machine (HPM 100, Bal-Tec/Leica). Frozen samples were transferred into a Leica EM AFS2 freeze-substitution system (Leica Microsystems, DE) precooled to −90 °C for substitution with acetone mixed with 5% (w/v) uranyl acetate in methanol for a final concentration of 0.5% (w/v) uranyl acetate, 2% (w/v) osmium tetroxide (OsO4), and 5% (w/v) water. The substitution was started at −90 °C and ended at 20 °C. Washing in acetone and embedding in Spurr resin (Polysciences, Chemie Brunschwig, CH) was done in a PELCO BioWave, Pro + microwave system (Ted Pella, USA), and the resin was polymerized at 60 °C for 48 h. Ultrathin sections (60 nm) were collected on formvar/carbon coated TEM grids (Quantifoil, DE), poststained with uranyl acetate and lead citrate and imaged in a Morgagni 268 (Thermo Fisher Scientific, USA) at an acceleration voltage of 100 kV in bright field mode.

### Starch extraction and quantification

Starch from whole rosettes of 35-day-old plants (harvested at the end of day) was extracted as previously described by Sharma et al. (2024) and quantified using an enzyme-based spectrophotometric assay as previously described by Hostettler et al. (2011).

### Extraction of proteins from leaves and immunoblotting

For the extraction of total leaf proteins, two 9-mm leaf discs of 5-week-old plants were snap-frozen in liquid nitrogen, homogenized using a Retsch mixer (Retsch MM301; 30 s, oscillation frequency set to 30 s^−1^, using two 4-mm glass beads per tube) and resuspended in Laemmli buffer (50 mm Tris-HCl, pH 6.8, 2% [w/v] SDS, 100 mm DTT, 3% [v/v] glycerol, and 0.005% [w/v] bromophenol blue), heated at 95 °C for 5 min and centrifuged for 1 min at 19,000 *g* at 20 °C. Total proteins present in the supernatant were loaded on SDS PAGE gels on an equal leaf area basis.

For the sequential extraction of soluble and membrane-bound proteins, two 9-mm leaf discs were homogenized in 200 µL ice-cold native protein extraction buffer (100 mm Tricine-KOH, pH 7.9, 5 mm MgCl_2_, complemented with protease inhibitor [Complete EDTA-free, from Roche]). Soluble proteins (denoted “S”) were recovered in the supernatant after spinning at 20,000 *g* for 5 min at 4 °C. The pellet was washed twice in ice-cold protein extraction buffer, with the second wash constituting the Triton-free wash (denoted “W”), then split into two fractions. The first pellet fraction was directly resuspended in Laemmli buffer, giving rise to the Triton-free insoluble protein extract (denoted “I”). The second pellet fraction was resuspended in native protein extraction buffer complemented with 1% (v/v) Triton X-100, and proteins present in the wash fraction (“W”, with Triton X-100) were recovered in the supernatant after centrifugation at 20,000 *g* for 5 min at 4 °C. The insoluble proteins in the pellet (“I”, with Triton X-100) were solubilized in Laemmli buffer. The soluble and wash fractions were also complemented with Laemmli buffer such that all fractions had been reconstituted in the same volume. Samples were heated at 95 °C for 5 min and centrifuged for 1 min at 19,000 *g* at 20 °C. Equal volumes of the supernatant were loaded on SDS PAGE gels.

Immunoblotting and near-infrared signal detection of IRDye 800CW secondary antibodies were conducted as previously described by Abt et al. (2020). The following primary antibody dilutions were used: 1:5000 diluted polyclonal anti-PsbW antibody from rabbit (Agrisera, catalog number AS05 060); 1:10,000 diluted monoclonal anti-actin antibody from mouse (Sigma-Aldrich, clone 10-B3 [MAbGPa]); 1:5000 diluted polyclonal anti-GFP antibody from rabbit (which also recognizes mCitrine; Torrey Pines Biolabs, catalog number TP401); 1:10,000 diluted anti-PsbA antibody from rabbit (Agrisera, catalog number AS05 084).

### Isolation of thylakoid membrane protein complexes and blue-native page

About 10 g of leaves from 35-day-old plants were homogenized in 150 mL extraction medium (50 mm 4-[2-hydroxyethyl]-1-piperazine ethanesulfonic acid [HEPES]-KOH, pH 7.8, 330 mm sorbitol, 2 mm EDTA, 1 mm MgCl_2_, 0.25% (w/v) bovine serum albumin [BSA], 50 mm sodium ascorbate) at 4 °C by two rounds of 3 s each in a Waring blender. The homogenate was filtered through 2 layers of nylon mesh 60 *µ*m pore size and centrifuged for 2 min at 2,000*g* and 4 °C. The pellet (containing chloroplasts) was carefully resuspended in 5 mL cold wash buffer (50 mm HEPES-KOH pH 7.8, 330 mm sorbitol) and loaded onto a two-step Percoll gradient, consisting of a bottom layer of 10 mL 85% Percoll (85% [v/v] Percoll, 50 mm HEPES-KOH pH 7.8, 330 mm sorbitol) and a top layer of 15 mL 40% Percoll (40% [v/v] Percoll, 50 mm HEPES-KOH, pH 7.8, 2 mm EDTA, 330 mm sorbitol, 2.1 mm MgCl_2_, 0.1% [w/v] BSA). After centrifugation for 10 min at 3750 *g* and 4 °C in a swing-out rotor, the interphase band containing intact chloroplasts was collected and washed once in 3 volumes of cold wash buffer to remove the Percoll (centrifugation for 5 min at 1,200 *g* for 4 °C). The pellet was resuspended 2 mL lysis buffer (10 mm Tris-HCl pH 8.0, 1 mm EDTA) and incubated on ice for 10 min. The released thylakoids were collected by centrifugation for 5 min at 2000 *g* and 4 °C, washed once in 15 mL resuspension buffer (10 mm HEPES-KOH pH 8.0, 5 mm MgCl_2_) and centrifuged for 5 min at 1,500 *g* and 4 °C. The pellet was resuspended in 1 mL resuspension buffer and thylakoids were normalized based on the chlorophyll content, quantified as described previously by Bals and Schünemann (2011). Thylakoid protein complexes were isolated by mixing the 80 µL thylakoid suspension with 320 µL solubilization buffer (50 mm Bis-Tris-HCl pH 7.0, 0.5 m aminocaproic acid, 10% [w/v] glycerol, 1% [w/v] digitonin, 1% [v/v] protease inhibitor cocktail for plant extracts [Amresco]) and incubating them on ice for 10 min. After centrifugation at 16,000 *g* for 10 min at 4 °C, the solubilized thylakoid proteins were recovered in the supernatant and kept on ice for immediate use.

For separation of thylakoid protein complexes by blue-native PAGE, 80 µL aliquots of the samples were supplemented with 2 µL CBB-G solution (5% w/v Coomassie Brilliant Blue G-250, 50 mm Bis-Tris-HCl pH 7.0, 0.5 m aminocaproic acid) and loaded with equal volumes on a 3% to 12% Bis-Tris mini protein gel (1 mm thick, Thermo Fisher Scientific). Blue-native PAGE was run with anode buffer (50 mm Bis-Tris, pH 7.0), and cathode buffer (200 mm Tricine, 15 mm Bis-Tris, 5% [w/v] Coomassie Blue G- 250) at 100 V for 3 h at 4 °C. The cathode buffer was then replaced by fresh buffer without Coomassie Blue G-250 and electrophoresis was continued for 3 h at 100 V at 4 °C.

### FIB-SEM imaging of isolated thylakoids

Freshly prepared, intact thylakoids were adhered on to Si-chips. The chips containing samples were immersed in 2.5% (w/v) glutaraldehyde in 0.3 m cacodylate buffer for 15 min and later washed in cacodylate buffer. Chip was further immersed in 1% (w/v) OsO4 for 15 min, washed in distilled water, and treated with graded series of ethanol. After final incubation in 100% pure ethanol, the chips were transferred to Critical Point Dryer (tousimis) for substituting ethanol against liquid CO2 with critical point adjusted at 31 °C and 73.8 bar. The pressure was released slowly, and chips were mounted with silver paint onto SEM-stubs, outgassed over-night and sputter coated with 4 nm Pt/Pd. The image acquisition was performed with Magellan 400 SEM. Helios 5 UX DualBeam system (Thermo Fisher Scientific) was used for FIB-milling (30 kV, 0.75 nA), and images were acquired (2 kV, 40 nA) with an Elstar in-column SE/BSE detector in immersion mode.

### Pulse-amplitude modulation measurements of chlorophyll fluorescence

Chlorophyll fluorescence in 14-day-old plants was analyzed using a FluorCam 800MF with FluorCam 7 software (both Photon Systems Instruments) and the default Quenching-1 program, with actinic light intensity set to 10% (approximately 150 *µ*mol photons m^−2^ s^−1^), saturation light intensity set to 100%, electronic shutter set to 1, and camera sensitivity set to 10.5%. Plants were subjected to 10-min dark adaptation immediately prior to the assessment.

### Photosynthetic gas exchange measurements

Leaf gas exchange dynamics of wild-type, *psbw*-1, and *psbw*-2 plants were measured using the LI-6800 infrared gas analyzer (LICOR Biosciences, USA). The cuvette environment was set to match the growth conditions at 20 °C and 65% relative humidity. Leaves in the vegetative growth stage, aged between 37 and 42 days, were mounted on a customized cuvette aperture of 1.13 cm^2^ to avoid potential chamber leakage. To further avoid systematic errors, range match, and warm-up tests were frequently conducted according to the LI-6800 user manual. All measurements were performed using an automatic program in the following sequence: (i) Dark-adapted F_v_/F_m_ measurement: before dawn, dark-adapted F_v_/F_m_ measurements were taken to assess whether the leaves were under stress. (ii) Light Response (A/Q) Curve: At dawn, leaves were acclimated for 30 min at 1,000 µmol photons m⁻² s⁻¹ and 400 ppm CO₂. Light intensities were then altered in a stepwise manner at the following levels: 1,000, 1,500, 2,000, 1,000, 700, 500, 350, 250, 150, 100, 80, 60, 40, and 0 µmol photons m⁻² s⁻¹. Measurements were recorded after 15 min at each light intensity, a tested duration to ensure steady-state CO₂ assimilation was reached. (iii) Intercellular CO_2_ concentration response (A/Ci) Curve: Following the A/Q curve, leaves were re-acclimated for 30 min at 1,000 µmol photons m⁻² s⁻¹ and 400 ppm CO₂. CO₂ concentrations were then adjusted stepwise to 400, 300, 250, 200, 150, 100, 50, 400, 600, 900, 1,200, and 1,500 ppm, with measurements taken after 15 min at each concentration. Rapid measurements at low CO₂ concentrations minimized changes in Calvin-Benson-Bassham cycle enzyme activation (Long 2003). The saturating light intensity of 1000 µmol photons m⁻² s⁻¹ was predetermined by pilot experiments.

Throughout the experiment, the light source consisted of 90% red light and 10% blue light; a minimum of 4 biological replicates were measured for each genotype. The key modeling parameter V_cmax_ (CO_2_-saturated maximum carboxylation rate of Rubisco), J_max_ (Light-saturated potential electron transport rate), and TPU (Triose phosphate utilization) were derived from the extended FvCB model (Farquhar et al. 1980; Yin et al. 2021) with the R package “Plantecophys” (Duursma 2015) for each replicate. During the FvCB model fitting, empirical values for the temperature responses of the kinetic constants K_c_, K_o_, and Γ* were used, as described in (Bernacchi et al. 2001; Medlyn et al. 2002). The g_m_ (Mesophyll conductance) was assumed to be infinite, as it is difficult to quantify precisely without the stable isotope method (Busch et al. 2020). We noted this assumption may negatively impact parameter estimations from FvCB models (Niinemets et al. 2009) despite *psbw*-2 mutants likely having altered g_m_ due to disorganized membranes. Eventually, a better model fitting was achieved with TPU limitation, as indicated by the average root-mean-square deviation (RMSE), which was lower when fitting with TPU limitation (7.36) compared to fitting without TPU limitation (7.57).

### Statistical analysis

A test for normality was performed on all data sets (Shapiro–Wilk test). The data points of the maximum quantum yield of PSII (F_v_/F_m_) data set did not show a normal distribution. Therefore, the Kruskal–Wallis test was used for statistical analysis. Starch content and fresh weight measurements were normally distributed. Statistical analyses were performed using one-way ANOVA and Dunnett's multiple comparison test. R 4.3.3 was used to conduct statistical analyses of gas exchange data; All other statistical analyses were performed using GraphPad Prism software.

### Accession numbers

The Arabidopsis genes studied in the present work have the following Arabidopsis Genome Initiative gene codes: AT2G30570 (*PsbW*), AT4G18240 (*SS4*) and AT3G16000 (*MFP1*). Protein identifiers of the *PsbW*orthologs used for phylogenetic analyses are given in Supplementary Fig. S1 and Supplementary Data Set 1.

## Supplementary Material

kiaf206_Supplementary_Data

## Data Availability

The data underlying this article are available in the article and in the online Supplementary Data Set S1.

## Supplementary data

The following materials are available in the online version of this article.

**Supplementary Table S1**. List of SNPs identified after NGS of line #144, which were located within genes and had a mutant allele frequency above 95%.

**Supplementary Table S2**. Primers used for genotyping.

**Supplementary Figure S1**. Maximum likelihood phylogenetic tree of putative PsbW orthologs (supporting Fig. 1E).

**Supplementary Figure S2**. Full size Immunoblot comparing the membrane association of PsbW and PsbW^G107R^ (supports Fig. 3A).

**Supplementary Figure S3**. Analysis of *psbw* mutant alleles and the F_1_ generation of their cross compared to the wild type.

**Supplementary Figure S4**. Confirmation of aberrant thylakoid phenotype in *psbw-*2.

**Supplementary Figure S5**. Number and morphology of starch granules in the double mutants *psbw*-2 *mfp1*-1 and *psbw*-2 *ss4*-1 (supporting Fig. 5A, B and C).

**Supplementary Figure S6**. NPQ measured on 5- to 6-weekold plants (*n* = 4 to 5).

**Supplementary Figure S7**. Image of 35-day-old plants of the *psbw* mutants and complementation lines compared to the Col-0 wild type.

**Supplementary Data Set 1**. Research data underlying the experiments presented in this study.

## Dive Curated Terms

The following phenotypic, genotypic, and functional terms are of significance to the work described in this paper:

FZL Gramene: AT1G03160
FZL Araport: AT1G03160

PsbW Gramene: AT2G30570
PsbW Araport: AT2G30570

At2g30570 Gramene: AT2G30570
At2g30570 Araport: AT2G30570

SS2 Gramene: AT1G74020
SS2 Araport: AT1G74020

TPU Gramene: Triose phosphate utilization
TPU Araport: Triose phosphate utilization

starch CHEBI: CHEBI:28017

MFP1 Gramene: AT3G16000
MFP1 Araport: AT3G16000

PTST2 Gramene: AT1G27070
PTST2 Araport: AT1G27070

MOS Gramene: malto-oligosaccharide
MOS Araport: malto-oligosaccharide

NCBI Gramene: https://www.ncbi.nlm.nih.gov
NCBI Araport: https://www.ncbi.nlm.nih.gov

NPQ Gramene: non-photochemical quenching
NPQ Araport: non-photochemical quenching

SS4 Gramene: AT4G18240
SS4 Araport: AT4G18240
