# Supplementary material for: A point mutation in the photosystem II protein PsbW disrupts thylakoid organization and alters starch granule formation
Source: Plant Physiol. 2025 May 29;198(2):kiaf206. doi: 10.1093/plphys/kiaf206 (PMC12124259; doi:10.1093/plphys/kiaf206)
Supplement: kiaf206_Supplementary_Data [file kiaf206_supplementary_data.zip › Ilseetal_SupplementaryMaterial.pdf]

Supplementary Table S1 List of SNPs identified after NGS of line #144, which were located within genes and had a mutant allele frequency above 95%. Sequencing depth represents the number of reads aligning to these sites. Predicted localization is the consensus location from SUBA5 (https://suba.live/).

Locus	Sequencing depth	Mutant allele count	Type of mutation	Name	Predicted localization
AT2G26640	115	100	synonymous	KCS11	plasma membrane
AT2G29990	127	126	synonymous	NDA2	mitochondrion
AT2G30330	105	103	missense	BLOS1	nucleus
AT2G30570	127	127	missense	PsbW	plastid

Supplementary Table S2 Primers used for genotyping.

Forward primer	Sequence	Reverse primer	Sequence	Purpose
PsbW fwd 1	TTGGAAAAGAGATGGAACGTG	PsbW rev 1	CTGAGACGTTTCCTTGCTTTG	genotyping *psbw*-1
T-DNA border primer SAIL_885_A03	GCCTTTTCAGAAATGGATAAATAGCCTTGCTTCC			
PsbW fwd 2	CTCCTCCTTATCAATGTTATGAC	PsbW rev 2	TTGGAAAAGAGATGGAACGTG	genotyping *psbw*-2
MFP1 fwd	CTCGGCAAGATACAAAAGCTG	MFP1 rev	GAGACCCGATCTCTAGATGCC	genotyping *mfp1*-1
T-DNA border primer SALK_124298	TGGTTCACGTAGTGGGCCATCG			
SS4 fwd	GGCACTGTTGAAGGTGAG	SS4 rev	CTGAATATGTGGAACCGGG	genotyping *ss4*-1
T-DNA border primer GABI_290D11			ATATTGACCATCATACTCATTGC	

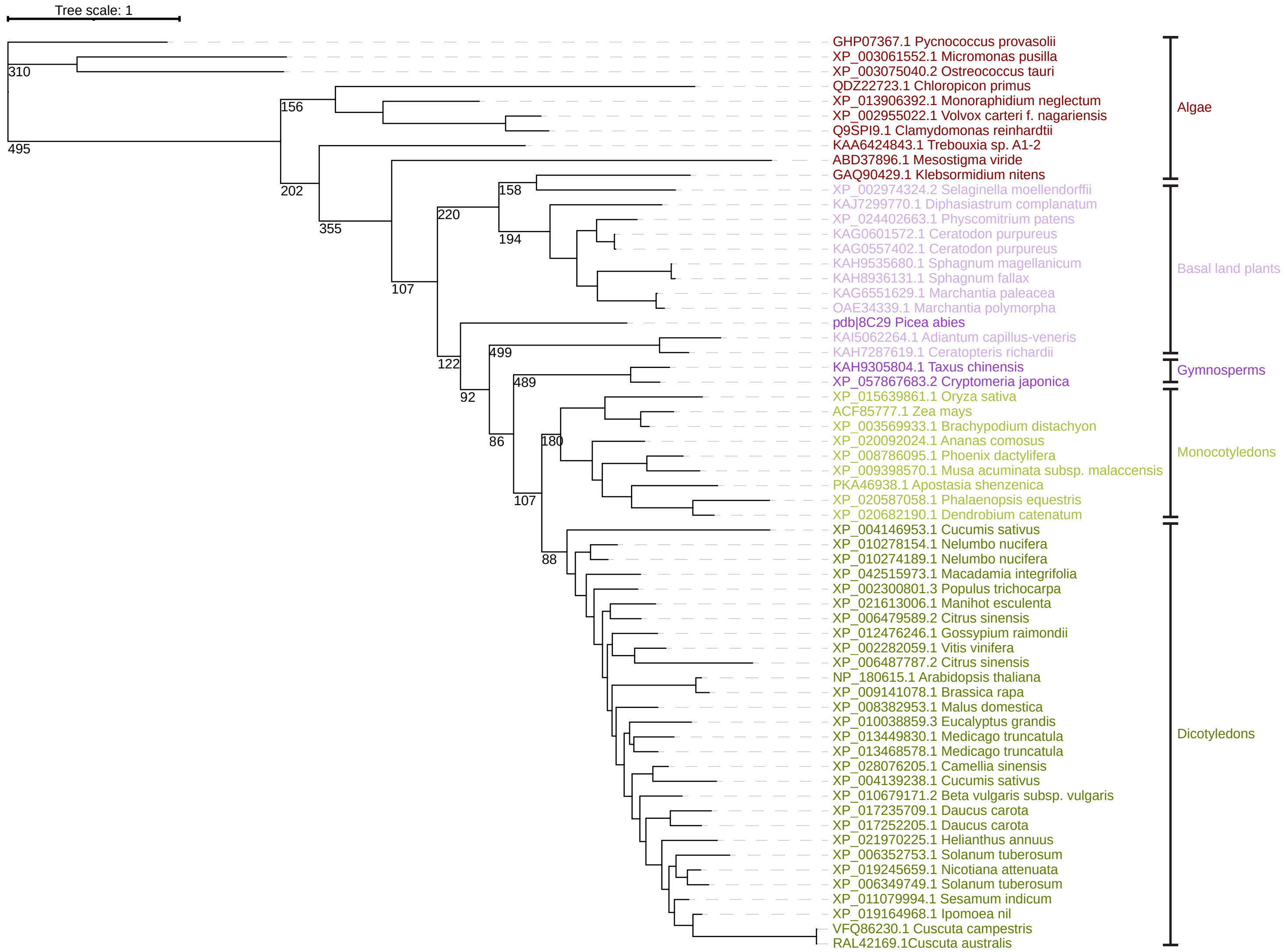

Supplementary Figure S1. Maximum likelihood phylogenetic tree of putative PsbW orthologs (supporting **Figure 1E**). The protein sequences under-lying the alignment are available in **Supplementary Dataset S1**. Branch lengths represent the number of amino acid substitutions per site (indicated by the scale bar), and bootstrap values (out of 500) are shown at some branch nodes.

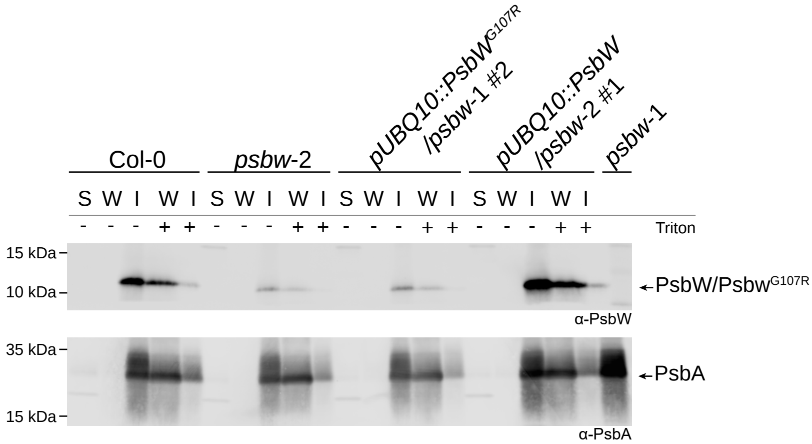

Supplementary Figure S2. Full size Immunoblot comparing the membrane association of PsbW and PsbW^{G107R} (supports **Figure 3A**). Proteins were extracted in native buffer without detergent, and the pellet after centrifugation washed either in the same buffer or in one containing 1% (v/v) Triton-X 100. Depicted are the soluble (S, prior to wash), wash (W) and insoluble (I, after wash) protein fractions treated with or without Triton-X 100. PsbA is a membrane-bound control protein. A total protein extract from *psbw*-1 (rightmost lane) served as a negative control.

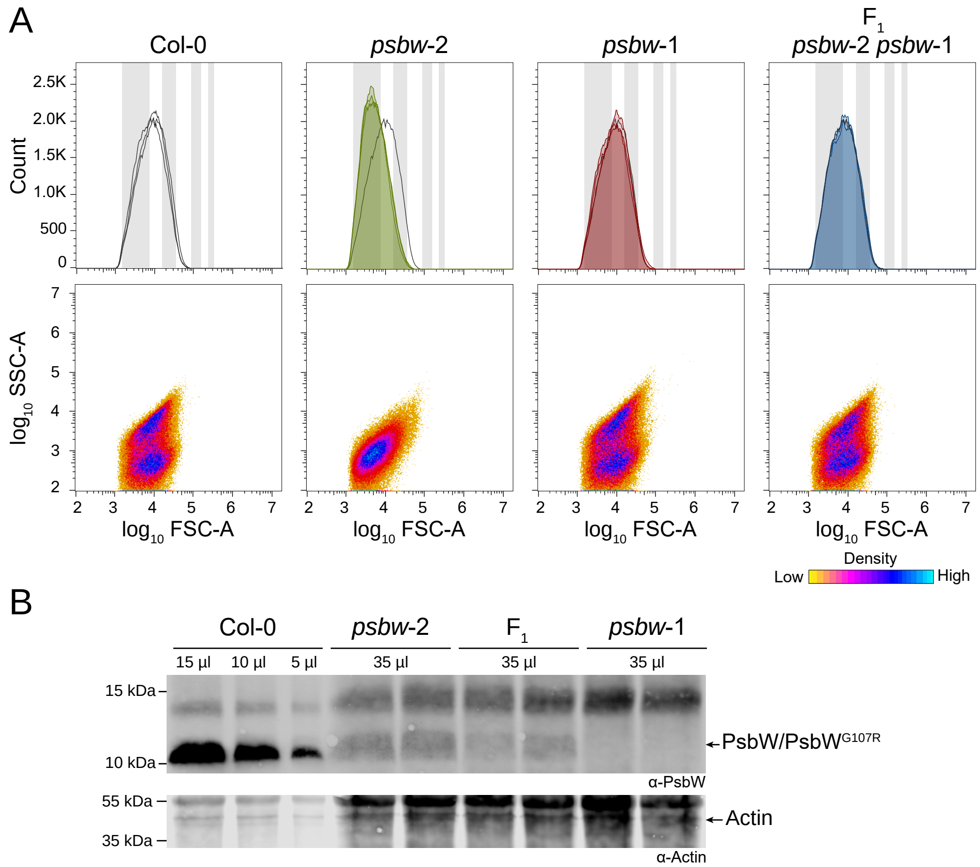

Supplementary Figure S3. Analysis of *psbw* mutant alleles and of the F_1 generation of their cross compared to the wild type. **A)** Flow cytometry analysis of leaf starch collected at the end of day is presented as described for **Figure 1** (*n* = 3 rosettes; 100,000 particles measured per biological replicate). Grey bars indicate the size ranges 0.1-0.5 µm, 1-2 µm, 4-6 µm, and 8-10 µm (left to right). A representative wild type (Col-0; *black line*) sample is included in each graph for comparison. The lower panels (SSC-A vs. FSC-A) indicate granule shape of a representative samples; data were consistent across the three biological replicates. **B)** Immunoblot of total leaf protein extracts using antibodies against PsbW (top panel) and actin (lower panel) as loading control. Amount of protein extract loaded is indicated above (100 µl corresponds to leaf disc with an area of 1.54 cm^2); for Col-0 three different amounts were loaded from the same extract. Two biological replicates are shown for *psbw*-2, F_1 and *psbw*-2.

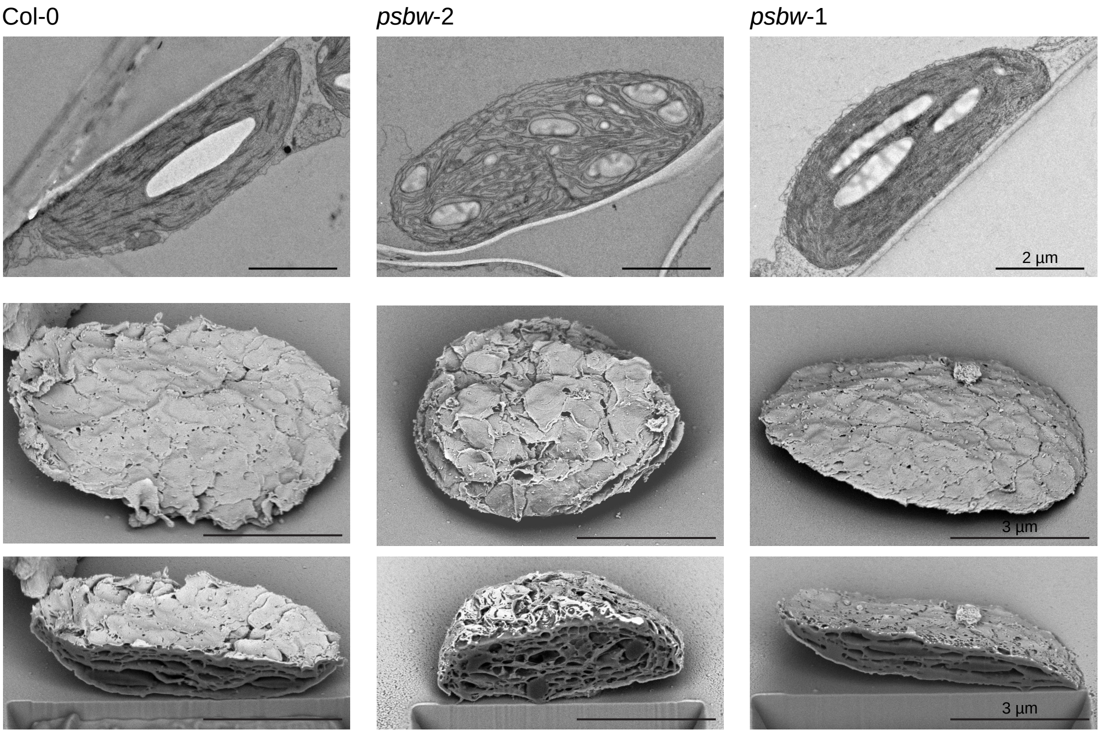

Supplementary Figure S4. Confirmation of aberrant thylakoid phenotype in *psbw*-2 by: Transmission electron micrographs of leaf sections harvested at the end of the day after high pressure freezing and freeze substitution (**top panels**); Scanning electron microscopy (SEM) of isolated thylakoids (**middle panels; lower panel**), and Scanning electron microscopy (SEM) of same isolated thylakoids after sectioning by focused ion beam milling. Scale bar units are shown in the last image of each row and apply to all images in that row.

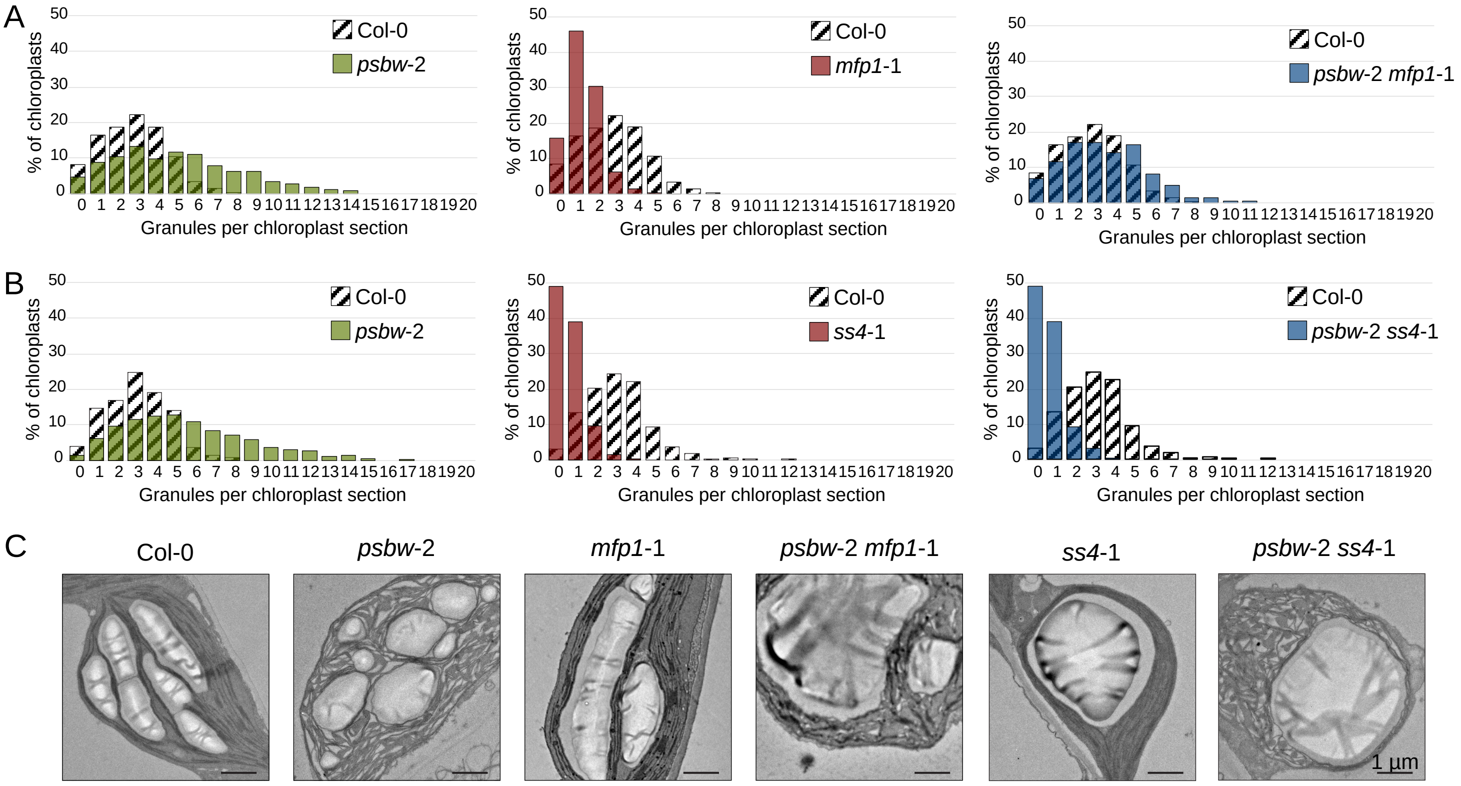

Supplementary Figure S5. Number and morphology of starch granules in the double mutants *psbw*-2 *mfp1*-1 and *psbw*-2 *ss4*-1 (supporting **Figure 5A**, **B** and **C**). **A**) Mutations in *psbw*-2 and *mfp1*-1 have additive effects on starch granule number (supporting **Figure 5A**). Histograms of starch granule count per chloroplast section of second biological replicates. Number of chloroplasts assessed: Col-0 wild type, 493; *psbw*-2, 456; *mfp1*-1, 509; *psbw*-2 *mfp1*-1, 206. **B**) The mutation in *ss4*-1 has a dominant effect over the mutation in *psbw*-2 on starch granule number (supporting **Figure 5B**). Histograms of starch granule count per chloroplast section of second biological replicates. Number of chloroplasts assessed: Col-0 wild type, 470; *psbw*-2, 365; *ss4*-1, 591; *psbw*-2 *ss4*-1, 374. **C**) Transmission electron micrographs of the same chemically fixed and embedded leaf sections as in **Figure 5C**. Scale bar units apply to all images in that row.

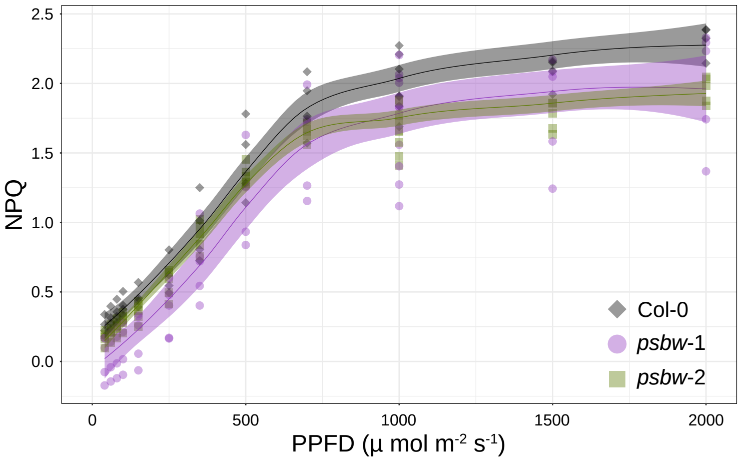

Supplementary Figure S6. NPQ measured on 5- to 6-week-old plants (*n* = 4 to 5). Dots show individual data points. Light shades around curves denote SE.

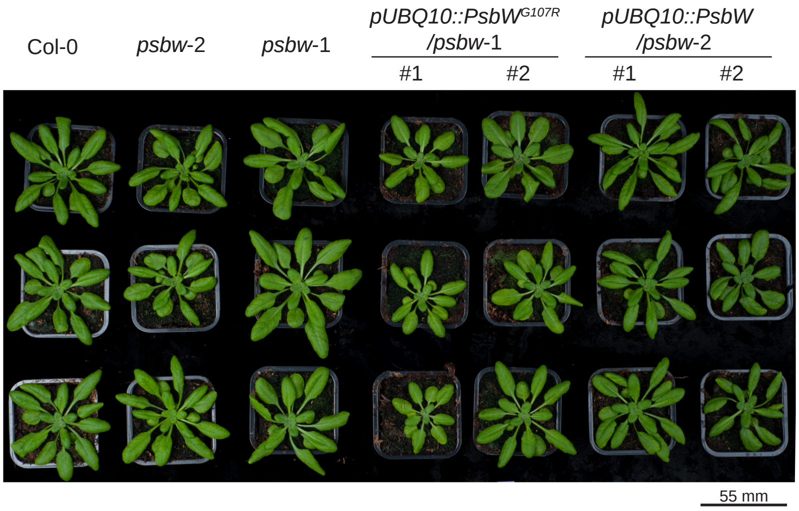

Supplementary Figure S7. Image of 35-day-old plants of the *psbw* mutants and complementation lines compared to the Col-0 wild type.
